# SIRT6 is an epigenetic repressor of thoracic aortic aneurysms via inhibiting inflammation and senescence

**DOI:** 10.1038/s41392-023-01456-x

**Published:** 2023-07-03

**Authors:** Yang-Nan Ding, Ting-Ting Wang, Shuang-Jie Lv, Xiaoqiang Tang, Zi-Yu Wei, Fang Yao, Han-Shi Xu, Yi-Nan Chen, Xiao-Man Wang, Hui-Yu Wang, He-Ping Wang, Zhu-Qin Zhang, Xiang Zhao, De-Long Hao, Li-Hong Sun, Zhou Zhou, Li Wang, Hou-Zao Chen, De-Pei Liu

**Affiliations:** 1 State Key Laboratory of Medical Molecular Biology, Department of Biochemistry and Molecular Biology, Institute of Basic Medical Sciences, Chinese Academy of Medical Sciences and Peking Union Medical College, Beijing, China; 2 Key Laboratory of Birth Defects and Related Diseases of Women and Children of MOE, State Key Laboratory of Biotherapy, West China Second University Hospital, Sichuan University, Chengdu, China; 3 National Health Commission Key Laboratory of Chronobiology, Development and Related Diseases of Women and Children, Key Laboratory of Sichuan Province, West China Second University Hospital, Sichuan University, Chengdu, 610041 China; 4 State Key Laboratory of Cardiovascular Disease, Fuwai Hospital, National Center for Cardiovascular Diseases, Chinese Academy of Medical Sciences and Peking Union Medical College, Beijing, China; 5 Medical Epigenetics Research Center, Chinese Academy of Medical Sciences, Beijing, China; 6 Center for Experimental Animal Research, Institute of Basic Medical Sciences, Chinese Academy of Medical Sciences and Peking Union Medical College, Beijing, China; 7 Beijing Key Laboratory for Molecular Diagnostics of Cardiovascular Diseases, Center of Laboratory Medicine, Beijing, China

**Keywords:** Cardiovascular diseases, Translational research, Epigenetics

## Abstract

Thoracic aortic aneurysms (TAAs) develop asymptomatically and are characterized by dilatation of the aorta. This is considered a life-threating vascular disease due to the risk of aortic rupture and without effective treatments. The current understanding of the pathogenesis of TAA is still limited, especially for sporadic TAAs without known genetic mutation. Sirtuin 6 (SIRT6) expression was significantly decreased in the tunica media of sporadic human TAA tissues. Genetic knockout of *Sirt6* in mouse vascular smooth muscle cells accelerated TAA formation and rupture, reduced survival, and increased vascular inflammation and senescence after angiotensin II infusion. Transcriptome analysis identified interleukin (IL)-1β as a pivotal target of SIRT6, and increased IL-1β levels correlated with vascular inflammation and senescence in human and mouse TAA samples. Chromatin immunoprecipitation revealed that SIRT6 bound to the *Il1b* promoter to repress expression partly by reducing the H3K9 and H3K56 acetylation. Genetic knockout of *Il1b* or pharmacological inhibition of IL-1β signaling with the receptor antagonist anakinra rescued *Sirt6* deficiency mediated aggravation of vascular inflammation, senescence, TAA formation and survival in mice. The findings reveal that SIRT6 protects against TAA by epigenetically inhibiting vascular inflammation and senescence, providing insight into potential epigenetic strategies for TAA treatment.

## Introduction

Thoracic aortic aneurysm (TAA) is a serious vascular disorder that can cause aortic rupture and sudden death. The incidence of TAA is estimated to be 7.6 per 100,000 persons per year, and the mortality rate of ruptured TAAs is as high as 70%.^1,2^ Owing to asymptomatic cases and difficulties in diagnosis, the reported rates are probably lower than the true rate of TAAs, and the incidence appears to be increasing over time.^3^ The current understanding of the pathogenesis of TAAs remains limited, and studies have mainly focused on genetic factors, including mutations in extracellular matrix components, canonical transforming growth factor-β signaling, and components or regulators of the vascular smooth muscle cell (VSMC) actomyosin cytoskeleton.^4,5^

VSMCs are important aortic components that play a vital role in maintaining the normal structure and function of the aorta. Unlike many other mature cell types, VSMCs do not differentiate terminally but retain a high degree of plasticity, which allows them to participate in diverse molecular events in the context of vascular diseases.^6,7^ Accumulating evidence reveals that VSMC dysfunction, caused by both genetic and non-genetic factors, contributes pivotally to the development and rupture of TAAs in humans and mice.^1,8^ However, only 20% of TAAs are attributed to specific genetic variants,^9^ and epigenetic mechanisms underlying VSMC dysfunction during the development and rupture of TAAs remain elusive.

The silent information regulator 2 protein (sirtuin) family is conserved deacetylase with remarkable abilities to inhibit disease and delay aspects of aging that relies on nicotinamide adenine dinucleotide (NAD^+^).^10^ Studies conducted in our and other laboratories have revealed that sirtuins play an essential role in cardiovascular homeostasis.^11–13^ Sirtuin 6 (SIRT6) is a histone deacetylase that promotes resistance to DNA damage and suppresses genomic instability, standing out as a key longevity protein in primates and mice.^14,15^ In the past decade, the function of SIRT6 in the development of disease has been clarified, with SIRT6 being implicated in many age-related disorders, including cancers, metabolic disorders, and cardio-cerebral vascular diseases.^16–18^ Studies by our group and others have revealed protective roles of SIRT6 in controlling inflammation and maintaining endothelial homeostasis and the VSMC phenotype in atherosclerosis and hypertension.^19–22^ However, the role of SIRT6 in the development of arterial aneurysms remains unknown.

In the present study, we investigated the role of SIRT6 in TAAs by using sporadic human TAA samples and experimental mouse models. In sporadic human TAA samples, expression of SIRT6 was significantly decreased and vascular inflammation and senescence were increased. In mice infused with angiotensin II (Ang II), *Sirt6* deficiency in VSMCs epigenetically upregulated the expression of the pivotal inflammatory factor interleukin (IL)-1β, which subsequently contributed to vascular inflammation, senescence and TAA formation, progression, and rupture.

## Results

### Reduction of SIRT6 expression in sporadic human TAA samples

**Fig. 1:**
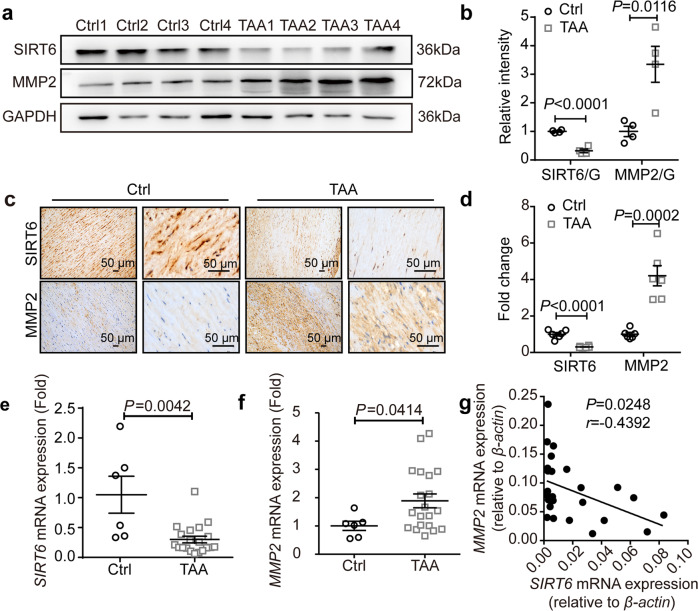
SIRT6 expression is decreased in sporadic human TAA samples. **a** Representative western blots of SIRT6 and MMP2 in sporadic human TAA and control (Ctrl) samples. **b** Normalized protein levels of SIRT6 and MMP2 in sporadic human TAA and Ctrl samples (*n* = 4). **c** Representative IHC staining images of SIRT6 and MMP2 in sporadic human TAA and Ctrl samples (scale bar: 50 μm). **d** Densitometric analysis of IHC staining for SIRT6 and MMP2 (*n* = 6). The results are normalized to the percentage of the stained area in Ctrl samples. **e**, **f** mRNA levels of *SIRT6* (**e**) and *MMP2* (**f**) in aortic homogenates from TAA patients (*n* = 20) and Ctrl samples (*n* = 6), as determined by quantitative reverse transcription-polymerase chain reaction (qRT-PCR). **g** Correlation between *SIRT6* and *MMP2* mRNA levels in 26 thoracic tissues. Each point in the scatter plot represents a single patient sample. Pearson’s correlation coefficient (r) and significant *P* values are shown

To investigate the potential roles of SIRT6 in sporadic TAA, we collected control thoracic aorta and TAA tissues to assess the expression of SIRT6. Elastica van Gieson (EVG) staining revealed strong and uniform elastin laminae in control samples, whereas disorganized elastin laminae with increased fragmentation (characteristic of aortic aneurysms) were prominent in samples from patients with sporadic TAA (Supplementary Fig. 1). Consistent with this, western blot results showed expression of pro-aneurysmal matrix metalloproteinase 2 (MMP2) to be significantly elevated in samples from patients with TAA, reflecting the severity of vascular wall matrix degradation. In contrast, there was a significant reduction in SIRT6 levels in samples from patients with TAA (Fig. 1a, b). Immunohistochemical (IHC) staining also revealed widespread SIRT6 expression in the tunica media of control aorta, but the SIRT6 levels were lower in samples from patients with TAA than in control samples (Fig. 1c, d). Furthermore, the reduction in *SIRT6* expression and increase in *MMP2* expression was verified at the transcriptional level in samples from patients with sporadic TAA (Fig. 1e, f), and there was a negative correlation between the mRNA levels of *SIRT6* and *MMP2* (Fig. 1g). Given that SIRT6 is an NAD^+^ - dependent deacetylase, we detected the NAD^+^ levels in TAA samples and the control thoracic aortas. The results showed that NAD^+^ levels were reduced in clinical TAA samples (Supplementary Fig. 2).

Collectively, the results demonstrate that SIRT6 expression is reduced in the tunica media in human TAA samples, suggesting that a reduction of SIRT6 levels in VSMCs may participate in the development of TAA.

### SMC-specific *Sirt6* deficiency promotes TAA formation and rupture in Ang II-induced mouse models

To establish a link between reduced SIRT6 expression in VSMCs and TAA formation, we generated conditional knockout mice in which *Sirt6* was deleted in VSMCs using the *Cre-Loxp* recombination system (Supplementary Fig. 3a–c). Arterial aneurysms were induced in mice with *Sirt6* deletion in VSMCs (S6-V-KO mice) and wild-type (WT) littermates by chronic infusion with Ang II (1.44 mg/kg/day) for 28 days, as we described previously.^13,23^ During the 28 days of Ang II treatment, 70.6% (36/51) of the S6-V-KO mice died of thoracic aorta rupture, with autopsy revealing blood clots in the thorax, and more than 60% of the surviving S6-V-KO mice had TAA (Supplementary Table 2.1).

**Fig. 2:**
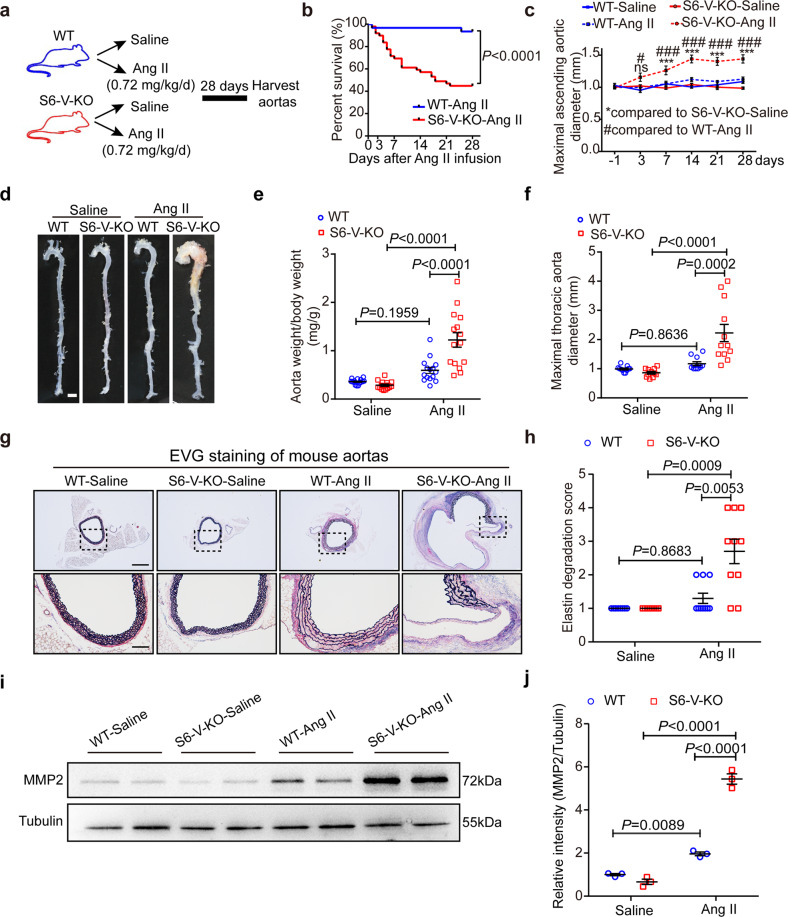
*Sirt6* deletion in VSMCs promotes TAA formation and rupture after Ang II infusion for 28 days. **a** Experimental design. S6-V-KO or WT mice were infused with saline or Ang II (0.72 mg/kg/d) for 28 days. **b** Survival curve after Ang II infusion (*n* = 31 for WT mice and *n* = 49 for S6-V-KO mice). **c** Maximal internal diameter of ascending aorta at days -1, 3, 7, 14, 21 and 28 (*n* = 12 mice/group). * and #, *P* < 0.05; ** and ##, *P* < 0.01; *** and ###, *P* < 0.001. **d** Representative images showing macroscopic features of the normal aorta and aneurysms in WT and S6-V-KO mice after infusion with saline or Ang II for 28 days (scale bar: 2 mm). **e**, **f** The ratio of aorta weight to body weight (*n* = 15 mice/group) (**e**) and the maximal thoracic aortic outer diameter (*n* = 11–12 mice/group) (**f**) in saline- and Ang II-infused mice at day 28. **g**, **h**. Representative images of Elastica van Gieson (EVG) staining of thoracic aortic sections from mice (**g**) and semiquantitative analysis of elastin degradation (**h**). Scale bars: 800 µm (top) and 200 µm (bottom). (*n* = 10 mice/group). **i** Representative western blots of MMP2. **j** Densitometric analysis of the protein levels of MMP2 (*n* = 3 mice/group)

The high lethality in S6-V-KO mice at this Ang II dose makes histological and etiological analyses difficult. We thus reduced the dose of Ang II to 0.72 mg/kg/day and infused S6-V-KO and WT mice with Ang II for 28 days (Fig. 2a). Mortality of S6-V-KO mice due to thoracic aorta rupture was significantly higher than that of WT mice from day 7 of Ang II infusion but not in the early stage (Fig. 2b). Approximately 55% (27/49) of S6-V-KO mice, but only 6.4% (2/31) of WT mice had died by day 28 (Fig. 2b and Supplementary Table 2.2). Few mice of either genotype died from abdominal aortic aneurysm (AAA) rupture. As shown in Supplementary Fig. 4a, neither Ang II treatment nor S6-V-KO affected the heart rate in mice. Ang II increased systolic and diastolic blood pressure, but there was no significant difference in the hypertensive response between the two genotypes (Supplementary Fig. 4b). We performed ultrasound imaging before and after Ang II infusion to monitor changes in the aortic diameter (Supplementary Fig. 4c). The diameter of the thoracic aorta, especially the ascending aorta, in the Ang II-infused S6-V-KO mice, increased progressively and was significantly larger than that in Ang II-infused WT mice (Fig. 2c and Supplementary Fig. 4d). Interestingly, the diameter of the abdominal aorta was comparable between the two genotypes (Supplementary Fig. 4d, e). More than half of the surviving Ang II-infused S6-V-KO mice were pathologically diagnosed with TAA (Fig. 2d and Supplementary Table 2.2). The extent of vascular lesions was assessed using in vitro measurements and dissection of the aorta from surviving saline- or Ang II-infused mice after euthanasia. The ratio of total aortic weight to body weight and the maximal thoracic aortic outer diameter were substantially higher in Ang II-infused S6-V-KO mice than in Ang II-infused WT mice and saline-treated control mice (Fig. 2e, f). Vascular remodeling occurred in almost all Ang II-infused S6-V-KO mice, as evidenced by an enlarged maximum vessel diameter and an increased ratio of vessel weight -to -body weight. EVG staining showed dramatic disruption and degradation of the medial elastic lamina in the thoracic aorta of S6-V-KO mice compared with results in WT mice after Ang II infusion (Fig. 2g, h and Supplementary Fig. 5a, b), consistent with a significant increase in MMP2 expression. (Fig. 2i, j). Loss of VSMCs is a hallmark of TAA,^24^ hence we examined the density of VSMCs in the aortic media using IHC staining α-smooth muscle actin. The results showed that VSMC density in S6-V-KO mice was lower than that in WT mice after Ang II infusion (Supplementary Fig. 5c, d).

Taken together, the findings reveal that *Sirt6* deficiency in VSMCs facilitates the development and rupture of Ang II-induced TAA.

### Vascular inflammation and senescence are enhanced in mouse TAA with *Sirt6* deficiency and in sporadic human TAA samples

**Fig. 3:**
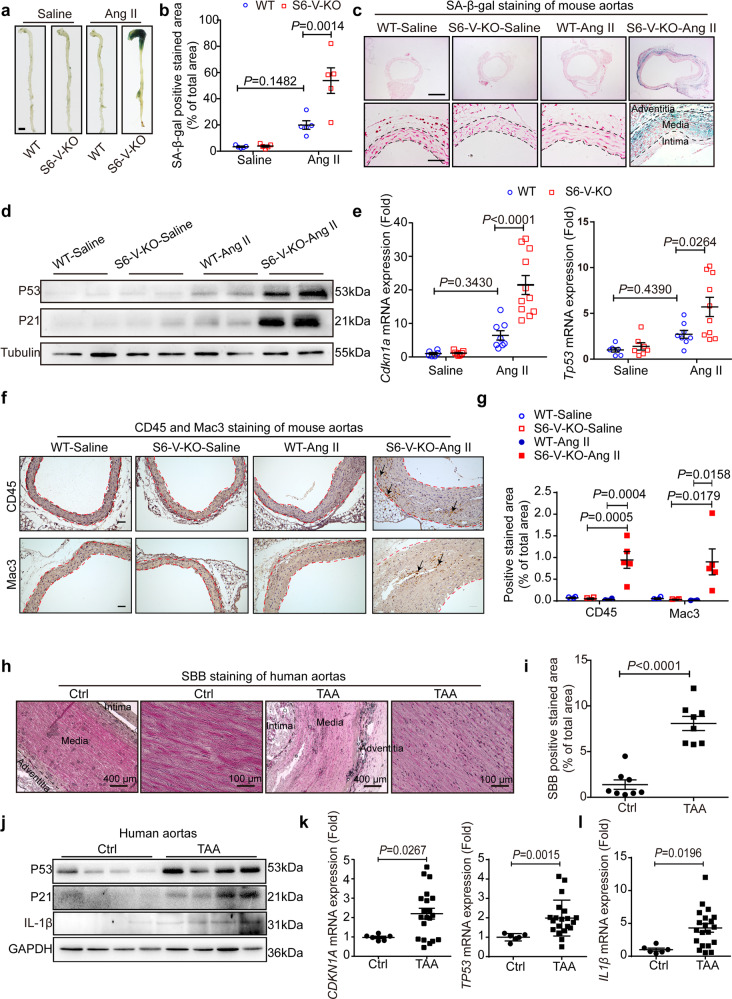
Increased vascular inflammation and senescence in Ang II-infused mice with *Sirt6* deficiency and human TAA samples. Mice were infused with saline or Ang II (0.72 mg/kg/d) for 28 days. **a** Representative images of SA-β-gal-stained aorta (scale bar: 2 mm). **b** Densitometric analysis of SA-β-gal staining in the whole aorta from WT and S6-V-KO mice infused with saline or Ang II for 28 days (*n* = 5 mice/group). **c** Representative images of SA-β-gal-stained transverse sections of thoracic aorta from WT and S6-V-KO mice infused with saline or Ang II. The blue regions are positively stained, and the nuclei were counterstained using nuclear Fast Red. Scale bars: 800 µm (top) and 100 µm (bottom). **d** Western blots of P21 and P53 in the mouse aorta. **e** mRNA levels of *Cdkn1a* and *Tp53* in the mouse aorta (*n* = 6–11 mice/group). **f** Representative images of IHC staining of leukocytes (CD45, top) and macrophages (Mac3, bottom) in the thoracic aortas of mice (scale bar: 50 µm). Arrows represent positively stained areas. **g** Statistical analysis of the CD45 and Mac3 positive area percentage in mouse aorta (*n* = 4–5 mice/group). **h** Representative staining with SBB as another indicator of cell senescence in human TAA and the control aorta. Scale bars: 400 µm (left) and 100 µm (right). **i** Statistical analysis of the SBB-positive area percentage in clinical TAA and control samples (*n* = 8). **j** Representative western blots of P21, P53 and IL-1β in human TAA and the control aorta. **k** The mRNA levels of *CDKN1A and TP53* in the human control thoracic aorta (*n* = 6) and TAA samples (*n* = 20). **l** The mRNA levels of *IL1β* in the human control thoracic aorta (*n* = 6) and TAA samples (*n* = 20)

Ang II infusion accelerates vascular senescence in vivo, which can be assessed by staining for senescence-associated β-galactosidase (SA-β-gal). Vascular senescence has recently been identified in aortic aneurysm diseases.^13,25^ We speculated that *Sirt6* deficiency in VSMCs might induce vascular senescence in mice after 28 days of Ang II infusion. To assess this, we performed SA-β-gal staining of the aorta from Ang II- or saline-infused S6-V-KO mice and littermate controls. Few SA-β-gal-positive areas were detected in saline-infused WT and S6-V-KO mice. Ang II infusion increased SA-β-gal-positive regions in the aorta of WT mice, whereas *Sirt6* deficiency in VSMCs led to further enlargement of SA-β-gal-positive staining areas (Fig. 3a, b). Furthermore, analysis of cross-sections showed that SA-β-gal-positive regions were mainly located in the tunica medium (Fig. 3c), showing that medial VSMC senescence is enhanced in the thoracic aorta of S6-V-KO mice after Ang II infusion for 28 days. Consistent with the SA-β-gal staining results, the aorta from S6-V-KO mice showed increased levels of the senescence markers P21 and P53 after Ang II infusion, as demonstrated by western blotting and qRT-PCR (Fig. 3d, e). In addition to vascular senescence, IHC staining demonstrated accumulation of inflammatory cells (macrophages and leukocytes) infiltrating the thoracic aorta of S6-V-KO mice after Ang II infusion for 28 days (Fig. 3f, g), revealing inflammation and senescence in the aorta of mice with *Sirt6* deficiency.

To further assess whether increased vascular inflammation and senescence also exist in sporadic human TAA samples, we used the histochemical dye Sudan Black B (SBB) to evaluate the content of lipofuscin, another hallmark of senescent cells in vivo.^26^ A significant number of positively stained granules (brown-to-black granules) were found in the samples from patients with TAA, but almost none were detected in the control samples (Fig. 3h, j). Western blotting and qRT-PCR consistently revealed a significant increase in vascular senescence biomarkers (P21 and P53), as well as the inflammatory factor, IL-1β,^24^ in TAA tissues compared with those in the control samples (Fig. 3j–l). Together, these observations indicate that vascular inflammation and senescence increase in mouse TAA with *Sirt6* deficiency and in human TAA samples.

### *Sirt6* deficiency increases vascular senescence in the late but not the early stage of TAA formation

**Fig. 4:**
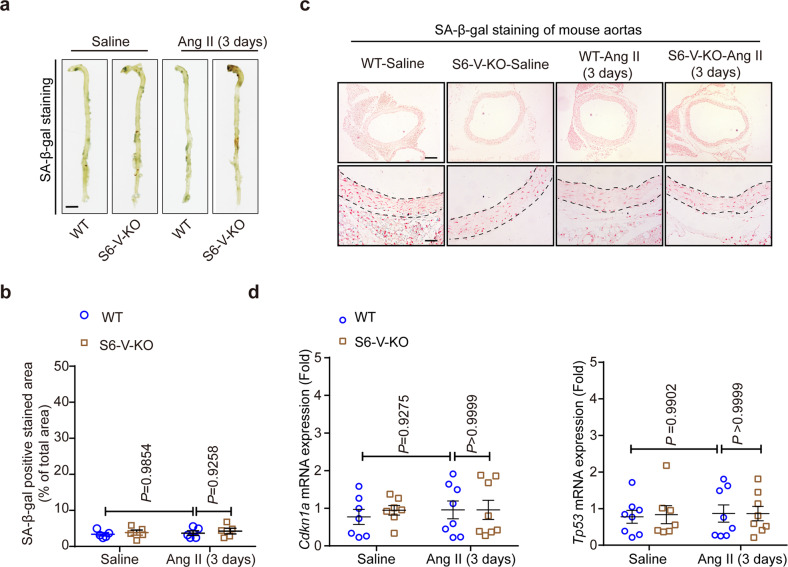
*Sirt6* deficiency does not induce vascular senescence in mouse aortas after Ang II infusion for 3 days. All mice were infused with saline or Ang II (0.72 mg/kg/d) for 3 days. **a** Representative images showing the SA-β-gal-stained aorta from the indicated groups (scale bars: 2 mm). **b** Densitometric analysis of SA-β-gal staining in whole aorta from WT and S6-V-KO mice infused with saline or Ang II (*n* = 5 mice/group). **c** Representative images of SA-β-gal-stained transverse sections of thoracic aortas from WT and S6-V-KO mice infused with saline or Ang II. Nuclei were counterstained using nuclear Fast Red. Scale bars: 400 µm (top) and 100 µm (bottom). **d** mRNA levels of *Cdkn1a* and *Tp53* in mouse aorta (*n* = 7–8 mice/group)

To better understand the mechanisms underlying the increase in vascular inflammation and senescence in TAA with VSMC-specific *Sirt6* deficiency, we compared vascular senescence between the late (day 28) and early (day 3) stages of TAA formation. While severe vascular senescence was observed after 28 days of Ang II infusion, SA-β-gal-positive regions were barely detected after Ang II infusion for 3 days, even in the S6-V-KO mice (Fig. 4a–c). Consistent with the results of SA-β-gal staining, mRNA levels of *Cdkn1a* and *Tp53* were comparable between Ang II-infused S6-V-KO mice and Ang II-infused WT mice (Fig. 4d). The increase in senescent aorta in the late stage, but not in the early stage, of TAA formation, suggests that other driving factors may precede occurrence of vascular senescence and deterioration in TAA.

### IL-1β expression is epigenetically increased by *Sirt6* deficiency in the early stage of TAA

**Fig. 5:**
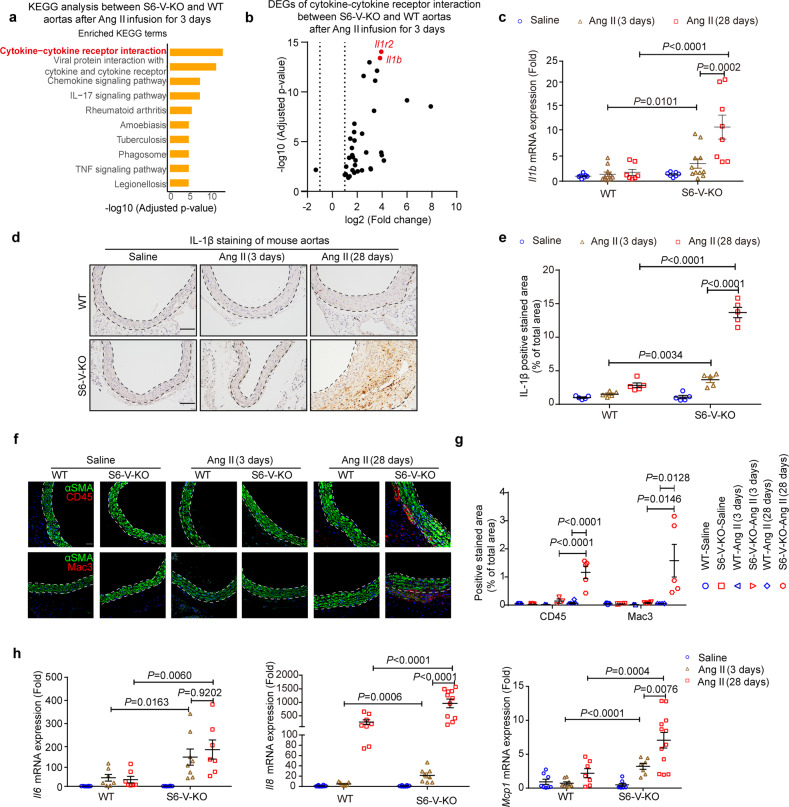
IL-1β expression is increased early in S6-V-KO mouse aorta after Ang II infusion for 3 days. **a** KEGG pathway analysis revealed that cytokine-cytokine receptor interaction was strongly affected in aorta of S6-V-KO mice compared with that in the aorta of WT littermates after Ang II-treated for 3 days (*n* = 3 mice/group). **b** Volcano plot of differentially expressed genes (DEGs) in the cytokine-cytokine receptor interaction pathway between S6-V-KO and WT mouse aortas after Ang II infusion for 3 days. The thresholds used for differential genes were a false discovery rate (FDR) of 0.05 and 1.5-fold up- or downregulation with *Sirt6* deficiency. **c** qRT-PCR analysis of *Il1b* expression in the mouse aorta (*n* = 7–11 mice/group). **d** Representative images of IHC staining of IL-1β in the mouse thoracic aorta (scale bar: 200 µm), with brown representing positive staining areas. **e** Densitometric analysis of IHC staining for IL-1β. The results were normalized to the percentage of the stained area in saline-treated WT mice. **f** Representative images of IF staining of leukocytes (CD45, top), macrophages (Mac3, bottom), VSMCs (αSMA) and nuclei (Hoechst) in the mouse thoracic aorta (scale bar: 100 µm). **g** Densitometric analysis of IF staining for CD45 and Mac3 (*n* = 3–6 mice/group). **h** qRT-PCR analysis of the expression of inflammation-related genes (*Il6*, *Il8*, and *Mcp-1*) in mouse aorta (*n* = 7–12 mice/group)

To further explore the potential pathological events and driving forces of vascular senescence in TAA, we performed transcriptome analysis of aorta from WT and S6-V-KO mice after Ang II infusion for 3 days. Kyoto Encyclopedia of Genes and Genomes (KEGG) enrichment analyses showed that *Sirt6* deficiency increased the expression of genes involved in several inflammation-related biological processes, particularly cytokine-cytokine receptor interactions (Fig. 5a). To identify the underlying cytokine signaling pathway that contributes to TAA formation with *Sirt6* deficiency, we analyzed the expression of these differential cytokines. *Il1r2* and *Il1b* were the genes with the most significant alterations in expression (Fig. 5b), but only upregulation of the expression of *Il1b*, the master cytokine of inflammatory vascular remodeling,^27^ was found to be in accordance with finding in clinical samples (Fig. 3l and Supplementary Fig. 6). Elevation of IL-1β expression in the aorta of Ang II-infused S6-V-KO mice received further experimentally support from the results of qRT-PCR and IHC staining after Ang II infusion for 3 days (Fig. 5c–e). Interestingly, immunofluorescence (IF) staining showed no inflammatory cells (macrophages and leukocytes) infiltrating the aorta of S6-V-KO mice after Ang II infusion in the early stage, but there was marked accumulation of inflammatory cells in the aorta after Ang II infusion for 28 days (Fig. 5f, g and Supplementary Fig, 7a), as shown in Fig. 3f. Notably, the aorta of S6-V-KO mice showed a further increase in the expression of IL-1β and related inflammatory genes, including *Il6*, *Il8*, and monocyte chemoattractant protein-1 (*Mcp-1*) after Ang II infusion for 28 days (Fig. 5c–e and Fig. 5h). *Sirt6* knockout was also found to promote the expression of inflammatory genes including *Il1b* in Ang II-treated VSMCs in vitro (Supplementary Fig. 7b). The findings suggest that IL-1β upregulation induced by *Sirt6* deficiency may contribute to subsequent pathological events, including vascular senescence and TAA formation.

Together, the observations indicate that IL-1β expression is increased in the early stage of TAA, and this increase may amplify vascular inflammation and senescence to facilitate the development of TAA.

### SIRT6 binds *Il1b* promoter to repress *Il1b* expression in mouse aortas

**Fig. 6:**
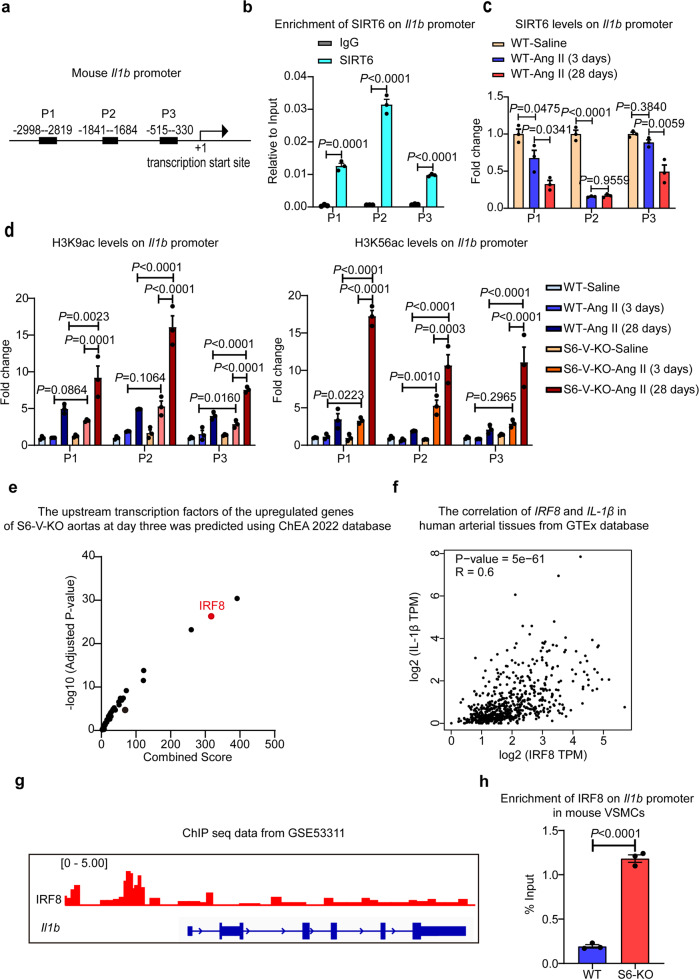
*Sirt6* deficiency increases H3K9ac and H3K56ac levels on the *Il1b* promoter. **a** Diagram showing designed primers for mouse *Il1b* promoter. **b** SIRT6 enrichment on the *Il1b* promoter as assessed by ChIP assays performed with chromatin prepared from aorta of WT mice (*n* = 3 mice/group). Chromatin was immunoprecipitated with normal rabbit IgG or antibodies against SIRT6, and precipitated genomic DNA was analyzed by real-time PCR using different primers for the different regions of the *Il1b* promoter. **c** SIRT6 enrichment on the *Il1b* promoter as assessed by ChIP assays performed with chromatin prepared from the WT mouse aorta with or without Ang II infusion for 3 days and 28 days (*n* = 3 mice/group). Procedures were as for (**b**). **d** ChIP assay of H3K9ac and H3K56ac at the *Il1b* promoter in the aorta of WT and S6-V-KO mice after saline or Ang II infusion for 3 days and 28 days (*n* = 3 mice/group). Chromatin was immunoprecipitated with normal rabbit IgG or antibodies against H3K9ac, H3K56ac and H3, and precipitated genomic DNA was analyzed by real-time PCR. **e** Upstream transcription factors of the upregulated genes in S6-V-KO aortas at day three were predicted using the ChEA 2022 database (https://maayanlab.cloud/Enrichr/). **f** The correlation of *IRF8* and *IL1β* in human aorta from the GTEx database. The Spearman analysis was performed using the webtool GEPIA (http://gepia.cancer-pku.cn/). **g** IRF8-binding to the *Il1b* promoter in mouse dendritic cells. The ChIP-seq data from GSE53311 was analyzed using IGV. **h** *Sirt6* knockout promotes IRF8 binding to *Il1b* promoter in VSMCs. The ChIP assay was performed with IRF8 antibody and qPCR of designated promoter region was performed (−1841 to −1648 bp)

We investigated potential mechanisms of how *Sirt6* deficiency regulates IL-1β expression. As a histone deacetylase, SIRT6 is capable of deacetylating histone H3 lysine 9 (H3K9) and 56 (H3K56).^19,28^ To test whether SIRT6 regulates IL-1β expression *via* an epigenetic mechanism, we performed chromatin immunoprecipitation (ChIP) with the mouse aorta after Ang II infusion for 3 days and 28 days. Three primers were designed to test the binding of SIRT6 to the *Il1b* promoter (Fig. 6a). The results showed that SIRT6 specifically binds to the *Il1b* promoter in the mouse aorta (Fig. 6b). Importantly, Ang II treatment reduced recruitment of SIRT6 to the *Il1b* promoter (Fig. 6c). We further performed ChIP followed by qPCR in the mouse aorta using antibodies against H3K9ac and H3K56ac to examine whether *Sirt6* deficiency affects the in vivo H3K9 and H3K56 acetylation levels on the *Il1b* promoter. Remarkably, *Sirt6* deficiency further enhanced H3K9ac and H3K56ac levels at different regions of the *Il1b* promoter, with further increases as the disease progressed (Fig. 6d). This finding provides evidence for an epigenetic mechanism underlying increased IL-1β expression in the aortas of Ang II-infused S6-V-KO mice.

To explore transcriptional factors bound to SIRT6-modified chromatin, we used the ChEA 2022 database to predict the upstream transcription factors for genes upregulated in S6-V-KO aortas at day 3 and identified interferon regulator factor 8 (IRF8) as a significantly enriched transcriptional factor (Fig. 6e). Using the GTEx database, we found that IRF8 and IL-1β had a positive correlation in the human aorta (Fig. 6f). We further analyzed the potential enrichment of IRF8 on the *Il1b* promoter using the previously reported ChIP-seq dataset GSE53311 (Fig. 6g). Consistent with our hypothesis, IRF8 binding peaks were enriched in the promoter regions of *Il1b*, suggesting that *Il1b* is regulated by the binding of IRF8. A ChIP assay in VSMCs also demonstrated that IRF8 bound to the *Il1b* promoter, and that was significantly enhanced by *Sirt6* knockout (Fig. 6h).

Taken together, the results indicate that SIRT6 directly binds to the *Il1b* promoter. *Sirt6* deficiency leads to hyperacetylation of H3K9ac and H3K56ac and enrichment of IRF8 on the *Il1b* promoter, promoting the transcription of *Il1b*.

### IL-1β contributes to *Sirt6* deficiency-mediated vascular inflammation, senescence, and TAA

**Fig. 7:**
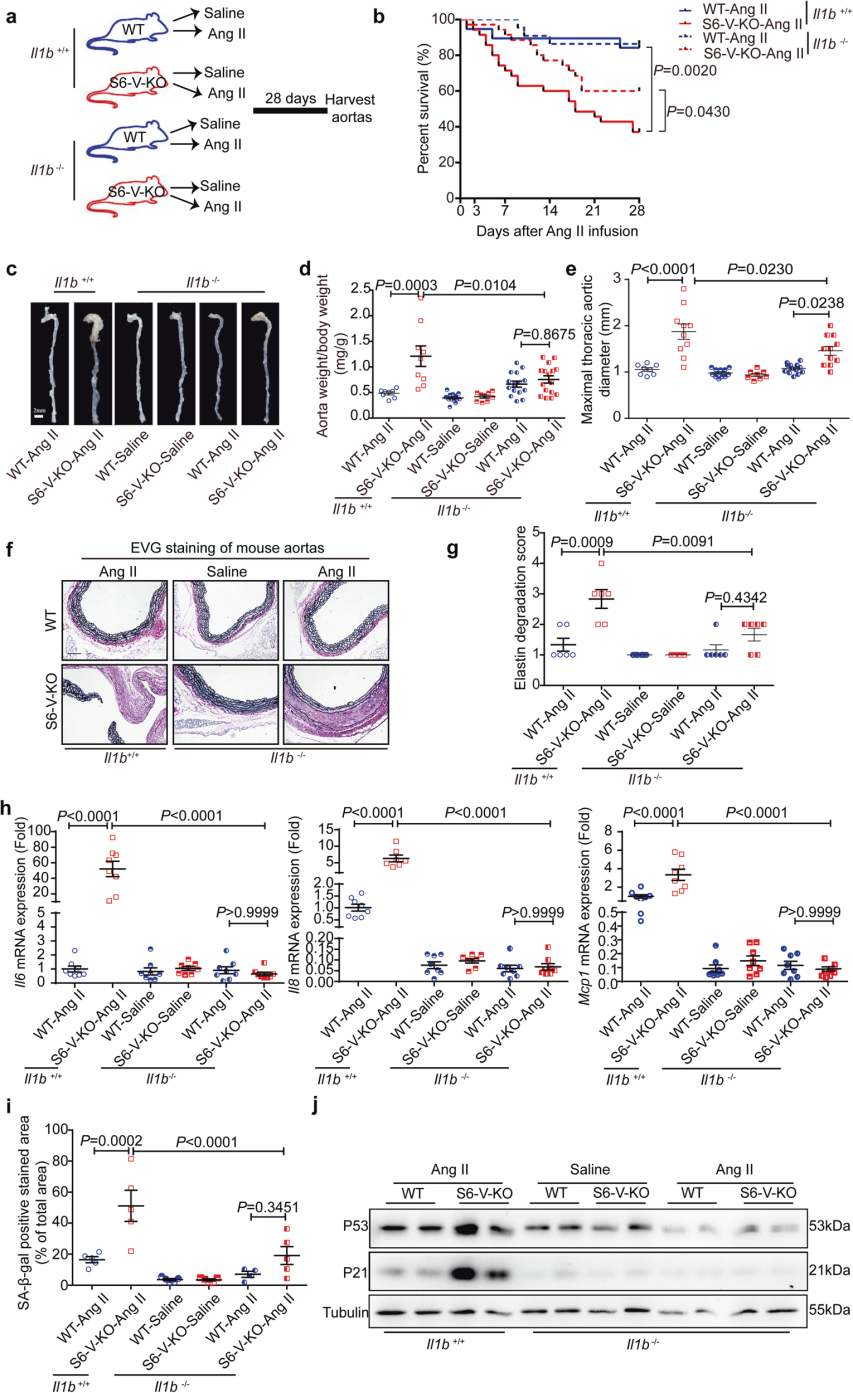
Genetic inhibition of *Il1b* alleviates Ang II-induced TAA and vascular inflammation and senescence in S6-V-KO mice. **a** Schematic of **e**xperimental design. All mice were infused with saline or Ang II for 28 days. **b** Survival curve of mice after Ang II infusion (*n* = 19 in the WT/*Il1b*^+/+^-Ang II group and *n* = 35 in the S6-V-KO/ *Il1b*^+/+^-Ang II group; *n* = 22 in the WT/ *Il1b*^−/−^-Ang II group, and *n* = 35 in the S6-V-KO/ *Il1b*^*−*/−^-Ang II group). **c** Representative images showing macroscopic features of the aorta (scale bar: 2 mm). **d**, **e** The ratio of aortic weight to body weight (*n* = 8–16 mice/group) (**d**) and the maximal thoracic aortic outer diameter (*n* = 8–13 mice/group) (**e**). **f**, **g** Representative images of EVG staining of mouse thoracic aortic sections (**f**) and semiquantitative analysis of elastin degradation (**g**). Scale bars: 200 µm (*n* = 6 mice/group). **h** mRNA levels of inflammatory genes in the mouse aorta (*n* = 7–8 mice/group). **i** Densitometric analysis of SA-β-gal-stained mouse aorta (*n* = 4–5 mice/group). **j** Protein levels of P21, P53 and Tubulin in aortic homogenate as determined by western blotting

To investigate the direct role of IL-1β in mediating vascular inflammation, senescence and TAA development with *Sirt6* deficiency in VSMCs, *Il1b*^−/−^ mice were crossed with S6-V-KO or WT mice to obtain S6-V-KO/*Il1b*^*−/−*^ and WT/*Il1b*^−/−^ mice (Supplementary Fig. 8). These animals, along with S6-V-KO/*Il1b*^+/+^ and WT*/Il1b*^+/+^ mice, were treated with Ang II for 28 days (Fig. 7a). *Il1b* knockout reduced the mortality and severity of TAA (Fig. 7b–g, Supplementary Fig. 9c, d, and Supplementary Table 2.3). Vascular inflammation was reduced in S6-V-KO mice by *Il1b* knockout after Ang II infusion for 28 days (Fig. 7h and Supplementary Fig. 9a, b). Moreover, *Il1b* knockout significantly inhibited vascular senescence in Ang II-infused S6-V-KO mice (Fig. 7i, j and Supplementary Fig. 10a, b).

**Fig. 8:**
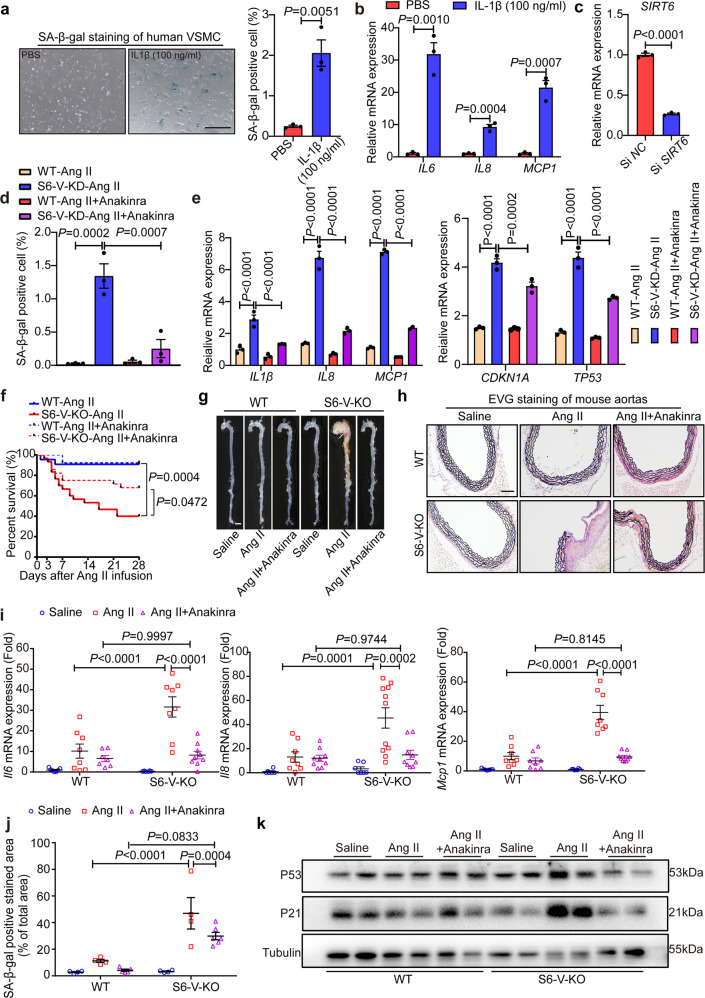
Pharmacological inhibition of IL-1β alleviates Ang II-induced inflammation, senescence and TAA in vitro *and* in vivo with *Sirt6* deficiency. **a** Representative images of SA-β-gal-stained human VSMCs with or without IL-1β (100 ng/ml) treatment and densitometric analysis of SA-β-gal positive cells. Blue-stained cells were considered senescent. Scale bar, 500 μm. **b** mRNA levels for inflammatory genes in human VSMCs (*n* = 3). **c** Relative mRNA levels of *SIRT6* in human VSMCs transfected with negative control siRNA (si *NC*) and *SIRT6* siRNA (si *SIRT6*) (*n* = 3). **d** Densitometric analysis of SA-β-gal-stained WT and *SIRT6* knockdown (S6-KD) human VSMCs with or without anakinra treatment following Ang II treatment and densitometric analysis of SA-β-gal- positive cells. (*n* = 3). **e** mRNA levels for inflammatory and senescence genes in WT and S6-KD human VSMCs with or without anakinra treatment after Ang II treatment (*n* = 3). **f** Survival curves for mice after Ang II infusion (*n* = 22 for WT-Ang II mice, *n* = 30 for S6-V-KO-Ang II mice, *n* = 13 for WT-Ang II-Anakinra mice and *n* = 29 for S6-V-KO-Ang II-Anakinra mice). **g** Representative images showing the macroscopic features of aorta (scale bar: 2 mm). **h** Representative images of EVG staining of the mouse thoracic aortic sections (Scale bars: 200 µm). **i** mRNA levels of inflammatory genes in the mouse aorta (*n* = 6–11 mice/group). **j** Densitometric analysis of SA-β-gal-stained mouse aorta (*n* = 4–6 mice/group). **k** Protein levels of P21, P53 and Tubulin in aortic homogenates as measured by western blotting

To further explore the contribution of IL-1β signaling to the function of SIRT6 in TAA, we treated WT human VSMCs and mouse VSMCs with recombinant IL-1β protein. IL-1β treatment increased the percentage of SA-β-gal positive cells in the human and mouse VSMCs (Fig. 8a and Supplementary Fig. 11a), and the inflammatory genes *IL6*, *IL8* and *MCP-1* also showed upregulated expression after IL-1β stimulation (Fig. 8b and Supplementary Fig. 11b). We found that *Mmp2* expression was increased after treatment with 100 ng/ml IL-1β (Supplementary Fig. 11c). However, SIRT6 may not increase *Mmp2* expression through deacetylated H3K9 and H3K56 on the *Mmp2* promoter (Supplementary Fig. 12a, b). The findings suggest that IL-1β signaling is sufficient to induce inflammation and senescence in human and mouse VSMCs.

Anakinra is a human IL-1 receptor (IL-1R) antagonist and functions as a competitive inhibitor of IL-1β binding.^29^ We tested whether anakinra inhibited vascular inflammation and senescence in vitro using human VSMCs. To examine whether anakinra can inhibit Ang II-induced inflammation and senescence, human VSMCs with or without *SIRT6* knockdown were treated with Ang II in the presence or absence of anakinra (Fig. 8c). Anakinra treatment reduced the expression of Ang II-induced senescence markers and inflammatory genes and blocked the effects of *SIRT6* knockdown in human VSMCs (Fig. 8d, e). The results suggest that inhibition of IL-1β by anakinra can protect against inflammation and senescence induced by *SIRT6* knockdown in vitro.

We also tested whether IL-1β signaling contributes to the effect of SIRT6 in vivo. To this end, Ang II-infused WT and S6-V-KO mice were treated with vehicle or anakinra at a dose of 35 mg/kg/day for 28 days. Anakinra treatment decreased Ang II-induced sudden death in S6-V-KO mice (9/29 *vs*. 18/30) (Fig. 8f and Supplementary Table 2.4). Furthermore, anakinra reduced the ratio and severity of TAA in the surviving S6-V-KO mice (Fig. 8g, Supplementary Fig. 9e, f and Supplementary Table 2.4). The ratio of aortic weight- to- body weight and the maximal aortic diameter were also reduced by anakinra in Ang II-infused S6-V-KO mice (Supplementary Fig. 13a, b). Additionally, better preserved elastic fibers were observed in the aorta of S6-V-KO mice treated with anakinra than in those of S6-V-KO mice treated with the vehicle (Fig. 8h and Supplementary Fig. 13c). We further examined whether anakinra ameliorated vascular inflammation and senescence in Ang II-induced S6-V-KO mice. The results showed that treatment with anakinra abolished the large increases in *Il6*, *Il8*, and *Mcp-1* expression, and the inflammatory cell infiltration in the arterial tissues of Ang II-infused S6-V-KO mice (Fig. 8i, and Supplementary Fig. 9a, b). Anakinra also inhibited vascular senescence in Ang II-infused S6-V-KO mice, as evidenced by reductions in the SA-β-gal-positive area and expression of P21 and P53 (Fig. 8j, k and Supplementary Fig. 13d, e).

In summary, the results reveal a critical effect of IL-1β signaling in vascular inflammation, senescence and TAA formation with *Sirt6* deficiency.

## Discussion

The current understanding of the pathogenesis of sporadic TAA is limited, and previous studies have mainly focused on genetic risk factors. In the present study, we identified the protective effects of the epigenetic enzyme SIRT6 on the development of TAA by using mouse genetic models and human TAA samples. This study has several major findings. First, a reduction in expression of the histone deacetylase SIRT6 was observed in the tunica media of sporadic clinical TAA samples. Second, *Sirt6* deficiency in VSMCs accelerated TAA formation and rupture in mice following treatment with varying doses of Ang II. Third, *Sirt6* deficiency epigenetically increased IL-1β expression in the early stage of TAA, facilitating vascular inflammation, senescence and the development of TAAs. Fourth, *Sirt6* deficiency-mediated aggravation of inflammation, senescence, TAA formation and survival was rescued by genetic inhibition (genetic knockout of *Il1b*) or pharmacological intervention in the IL-1β signaling pathway (inhibition of IL-1β binding to IL-1R), indicating that inhibition of IL-1β signaling contributes to SIRT6 function. The findings indicate that epigenetic factors are crucial for vascular homeostasis and the development of TAA.

Although SIRT6 has been implicated in some age-related vascular diseases,^18–20,22^ the underlying mechanisms of how endogenous SIRT6 prevents vascular diseases remain not fully understood. We found that *Sirt6* deficiency in VSMCs led to increased vascular inflammation, senescence and the development of TAA; however, *Sirt6* deficiency increased IL-1β expression but not systematic inflammation and vascular senescence in the early stage of Ang II-induced TAA. The continuous increase in IL-1β expression resulting from *Sirt6* deficiency may further lead to recruitment of inflammatory cells and vascular inflammation and senescence in the late stage of TAA. Increased inflammation was consistently associated with cellular senescence in sporadic human TAA samples. Importantly, inhibition of IL-1β by pharmacological or genetic approaches inhibited vascular inflammation, senescence, and TAA formation in S6-V-KO mice. The results suggest that SIRT6 regulates the inflammatory factor IL-1β in VSMCs, contributing to the prevention of TAA. During aging, a chronic, sterile, low-grade inflammation referred to inflammaging develops and contributes to the pathogenesis of age-related diseases.^30^ Our findings reveal a previously unrecognized role for SIRT6 in preventing the development of TAA by inhibiting IL-1β and suggest that targeting inflammation not senescent cells^31^ can rescue age-related diseases, an approach that may be most effective at an early stage of health decline. The results provide new insights into the anti-inflammaging strategy for the prevention of sporadic TAA. Previous studies have revealed that inflammasomes and cleaved IL-1β contribute to the development of TAA and other vascular diseases, including atherosclerosis.^24,27,32^ Our findings identify SIRT6 as an epigenetic factor controlling IL-1β at the transcriptional level, enriching our understanding of IL-1β regulation and revealing an inflammasome-independent role for IL-1β in aging-related diseases such as TAA.

A critical hallmark of TAA progression is fragmented elastic laminae in the tunica media, which correlate with elevated levels of MMPs,^33^ especially MMP2 and MMP9. In elastase-induced thoracic aorta, genetic and pharmacological inhibition of IL-1β decreased MMP9 expression.^24^ Our results showed that IL-1β and MMP2 levels were increased in both human and mouse TAA samples. Genetic and pharmacological inhibition of IL-1β inhibited MMP2 upregulation in TAA, however, SIRT6 did not directly regulate *Mmp2* expression by influencing the acetylation of H3K9 and H3K56 on the *Mmp2* promoter, but it may act in an IL-1β dependent manner.

Different embryonic origins, cell biology, anatomical structures, and hemodynamics of the thoracic and abdominal aortas lead to different lesions.^9^ We found that Ang II infusion promoted the formation of aneurysms in the thoracic aorta, but not in the abdominal aorta, in S6-V-KO mice. Further studies are required to investigate the different roles of SIRT6 in TAAs and AAAs.

Senescent VSMCs have been reported to accumulate in atherosclerotic plaques in mice and humans.^34,35^ Our previous study demonstrated a causal role for senescent VSMCs in the development of mouse AAAs.^13^ In this study, we provided evidence that cellular senescence occurs in human TAAs and is correlated with the expression of the vascular inflammation orchestrator IL-1β. In Ang II-induced mouse models, VSMC *Sirt6* deficiency led to vascular inflammation and subsequent senescence, consistent with the finding that SIRT6 prevents cells from premature senescence in the vascular system.^20,36^ Our study identified a complementary mechanism underlying SIRT6-mediated prevention of cell senescence by targeting IL-1β. Together with the findings from other groups,^37,38^ the results suggest that IL-1β signaling may serve as a driver of inflammation-associated senescence in VSMCs. However, inhibiting IL-1β signaling (*Il1b* knockout or pharmacological inhibition of IL-1β) only partially ameliorated the effect of *Sirt6* deficiency in TAAs. SIRT6 is a multitasking factor involved in DNA damage response, telomeric maintenance, oxidative stress, inflammation, and cellular metabolism.^39^ In addition to deacetylating H3K9ac and H3K56ac on the *Il1b* promoter, other regulatory functions of SIRT6 in inflammation and senescence have been demonstrated. Our previous study showed that SIRT6 can inhibit aortic inflammation by regulating H3K9ac and H3K56ac on NKG2D ligand gene promoters in macrophages.^19^ Endothelial SIRT6 has been shown to deacetylate H3K9ac to prevent endothelial senescence.^22^ SIRT6 also can regulate telomeric H3K9ac deacetylation and protect against telomere damage-associated senescence and inflammation.^20^ These studies indicate that SIRT6 plays a crucial role in regulating inflammation and senescence, and the same mechanisms may also be involved in TAA formation.

Epigenetic mechanisms are fundamental in cardiovascular homeostasis, and inhibitors or activators of epigenetic enzymes have shown promise for the treatment of cardiovascular diseases including atherosclerosis and heart failure.^40,41^ Growing evidence has demonstrated that SIRT6 functions as a deacetylase, mono-ADP-ribosyltransferase and long fatty deacylase and participates in a variety of cellular signaling pathways from DNA damage repair in the early stage to disease progression.^39^ However, TNFα, a fatty acylated protein,^42^ showed no statistical difference in the serum of our model mice (data not shown), supporting a crucial role for SIRT6 deacetylase activity in TAA. Vascular inflammation, senescence, and the occurrence and development of TAA may be further verified in *Sirt6* conditional point mutant knock-in mice in future work. Meanwhile, the role of SIRT6 multi-enzymatic function on TAA still needs further study. Our previous work showed that activation of the histone deacetylase SIRT1 by caloric restriction represses the development of arterial aneurysms, and SIRT1 activators have been tested for the potential to treat vascular diseases in animals and humans.^23,43^ More than ten SIRT6 activators (*e.g*., MDL-811) have been developed, some of which have been shown to significantly affect aging and stroke.^44,45^ Given the pivotal role of SIRT6 in aging and related vascular diseases, the potential of SIRT6 activators to treat TAA and other vascular diseases, such as atherosclerosis, should be explored.

## Materials and methods

### Human aortic samples

All protocols using human specimens were approved by the Research Ethics Committee of Fuwai Hospital and the Research Ethics Committee of the Institute of Basic Medical Sciences, Chinese Academy of Medical Sciences and Peking Union Medical College (ethical codes YZW16003, 2020010 and 009-2014). In this study, aortic specimens from patients who underwent surgery of the ascending aorta after the development of TAAs were used. Patients with a familial history and aneurysm-related gene mutations (including mutations in *FBN1, TGFB2, SMAD3, TGFBR1, TGFBR2, ACAT2, MYH11, SMAD4, MYLK, NOTCH1, PRKG1, SKI, COL3A1, SLC2A10*, and *FBN2*) were excluded. Control specimens of the ascending aorta were obtained from donor bodies with no evidence of aortic disease or valvular malformation. Additional information is provided in Supplementary Table 1.

The human samples were washed three times using PBS, divided into small pieces and immediately frozen in liquid nitrogen after being isolated from the patients. The samples were stored in liquid nitrogen before use. When needed, they were removed and ground using a mortar and pestle. Subsequently, reagents were added to extract proteins and RNA separately for further analysis.

### Animal experiments

All animal treatments and experimental protocols were approved by the Animal Care and Use Committee at the Institute of Basic Medical Sciences, Chinese Academy of Medical Science and Peking Union Medical College. Mice were maintained on a 12:12 h day: night cycle and provided constant access to food and water.

We established *Sirt6*-VSMC-specific knockout (S6-V-KO) mice using the *Cre/Loxp* strategy. *Sirt6*^*flox/flox*^ mice expressing *Sirt6*^exon2-3^ flanked by *LoxP* sites on a 129/SV background were purchased from The Jackson Laboratory (stock number: 017334). *SM22α-Cre*^*+/−*^ mice on a 129/SV background with a *Cre recombinase* gene inserted into the endogenous *transgelin* (*SM22α*) locus were purchased from The Jackson Laboratory (stock number: 006878). Mice on the 129/SV background were backcrossed with mice on the C57BL/6 J background for at least 10 generations to yield *Sirt6*^*flox/flox*^ and *SM22α-Cre*^*+/−*^ mice on the C57BL/6 J background. *Sirt6*^*flox/flox*^ mice on the C57BL/6 J background were crossed with *SM22α-Cre*^*+/−*^ mice on the C57BL/6 J background, to generate S6-V-KO mice. The F1 progeny (*SM22α-Cre*^*+/−*^; *Sirt6*^*flox/+*^ mice) were backcrossed with *Sirt6*^*flox/flox*^ mice to fix the *Sirt6*^*flox/flox*^ genotype and obtain *SM22α-Cre*^*+/-*^; *Sirt6*^*flox/flox*^ mice. Finally, male *SM22α-Cre*^*+/-*^; *Sirt6*^*flox/flox*^ mice of a C57BL/6 J background were crossed with female *Sirt6*^*flox/flox*^ mice on the C57BL/6 J background to generate S6-V-KO mice (*SM22α-Cre*^*+/−*^; *Sirt6*^*flox/flox*^) and WT littermates (*Sirt6*^*flox/flox*^) as controls. Successful deletion of *Sirt6* in VSMCs in vivo was confirmed by western blot analysis.

*Il1b*^−/−^ mice of the C56BL/6 J background were purchased from Shanghai Model Organisms, China. We generated S6-V-KO/*Il1b*^*−/−*^ mice by crossing S6-V-KO mice with *Il1b*^*−/−*^ mice. First, we crossed male S6-V-KO (*SM22α-Cre*^*+/−*^; *Sirt6*^*flox/flox*^; *Il1b*^+/+^) mice (C57BL/6 J background) with female *Il1b*^*−/−*^ (*SM22α-Cre*^*−/−*^; *Sirt6*^*+/+*^; *Il1b*^−/−^) mice (C57BL/6 J background) to obtain F1 progeny (1/2 ratio of *SM22α-Cre*^*+/−*^; *Sirt6*^*flox/+*^; *Il1b*^+/−^ mice and 1/2 ratio of *SM22α-Cre*^*−/−*^; *Sirt6*^*flox/+*^; *Il1b*^+/−^ mice). Male F1 progeny (*SM22α-Cre*^*+/−*^*; Sirt6*^*flox/+*^*; Il1b*^*+/−*^ mice) were crossed with female F1 progeny (*SM22α-Cre*^*−/−*^; *Sirt6*^*flox/+*^; *Il1b*^+/−^ mice) to obtain F2 progeny, and male *SM22α-Cre*^*+/−*^; *Sirt6*^*flox/flox*^; *Il1b*^−/−^ mice and female *SM22α-Cre*^*−/−*^; *Sirt6*^*flox/flox*^; *Il1b*^−/−^ mice were crossed to generate S6-V-KO/*Il1b*^*−/−*^ (*SM22α-Cre*^*+/−*^; *Sirt6*^*flox/flox*^; *Il1b*^−/−^) mice and WT/*Il1b*^*−/−*^ (*SM22α-Cre*^*−/−*^; *Sirt6*^*flox/flox*^; *Il1b*^*−/−*^) mice.

All mice were genotyped by PCR using toe-clip samples. The primers used are listed in Supplementary Table 3.

### Mouse model of TAA and anakinra treatment

Eight- to twelve-week-old male mice fed a normal chow diet were used in this study. We used an Ang II-induced aneurysm model to assess the effect of Sirt6 deficiency on TAA development. Mice were subcutaneously infused with Ang II (Sigma-Aldrich, Cat No. A9525) at a dose of 1.44 or 0.72 mg/kg/d via osmotic pumps (Alzet, model 2004) for 28 days or osmotic pumps (Alzet, model 2001) for 3 days. The mice in the control group received saline using the same method. The detailed procedure was performed as previously described.^13^ Briefly, Ang II was dissolved in sterile saline and infused using Alzet osmotic pumps. The mice were anesthetized with an intraperitoneal injection of tribromoethanol. Pumps were inserted into the subcutaneous space of anesthetized mice by making a small incision in the back of the neck, which was closed with sutures. The incision site healed rapidly without any infection in all mice. Anakinra was delivered by an osmotic pump at a dose of 35 mg/kg/d at the same time as Ang II.

### Statistical analysis

All experiments were performed at least in triplicate unless otherwise stated. Homogeneity of the variance was assessed via the F test (2 groups) or Brown–Forsythe test (≥3 groups). The normality of the data was assessed via the Shapiro–Wilk test. The survival of the mice was analyzed by the log-rank (Mantel–Cox) test. When reporting 2 groups with normal distribution, we used the standard Student’s *t* test for equal variance or the Welch *t* test for unequal variance. The Mann–Whitney test was used when a normal distribution could not be confirmed. For comparison of more than two groups, two-way ANOVA followed by the Bonferroni *post hoc* test was applied for multiple comparisons when the assumptions (equal variances and normal distribution) were satisfied. The *P* values were adjusted for multiple comparisons where appropriate. *P* values of <0.05 were considered statistically significant. All statistical analyses were carried out using GraphPad Prism 6.0 software. The quantitative results are expressed as the mean ± SEM.

An expanded materials and methods section is available in the Supplementary Materials.

## Supplementary information

Supplementary Material

## Data Availability

The authors declare that all the data supporting the findings of this study are available within the article and its Supplemental information files. All materials in this article are available upon reasonable request from the corresponding authors.
