# Supplementary material for: SIRT6 is an epigenetic repressor of thoracic aortic aneurysms via inhibiting inflammation and senescence
Source: Signal Transduct Target Ther. 2023 Jul 3;8:255. doi: 10.1038/s41392-023-01456-x (PMC10315397; doi:10.1038/s41392-023-01456-x)
Supplement: Supplementary file 1 — Supplementary Material [file 41392_2023_1456_MOESM1_ESM.docx]

**Supplementary Materials for**

**SIRT6 is an Epigenetic Repressor of Thoracic Aortic Aneurysms via Inhibiting Inflammation and Senescence**

Yang-Nan Ding^1#^, Ting-Ting Wang^1#^, Shuang-Jie Lv^1#^, Xiaoqiang Tang^2, 3^, Zi-Yu Wei^1^, Fang Yao ^4^, Han-Shi Xu^4^, Yi-Nan Chen^4^, Xiao-Man Wang ^1^, Hui-Yu Wang^1^, He-ping Wang^1^, Zhu-Qin Zhang^1, 7^, Xiang Zhao^1^, De-Long Hao^1^, Li-Hong Sun^5^, Zhou Zhou^4,6^, Li Wang^4, 7*^, Hou-Zao Chen^1, 7*^, De-Pei Liu^1, 7*^

Correspondence to: [liudp@pumc.edu.cn](mailto:liudp@pumc.edu.cn); [chenhouzao@ibms.cams.cn](mailto:chenhouzao@ibms.cams.cn);

[wangl@pumc.edu.cn](mailto:wangl@pumc.edu.cn)

**The PDF file includes:**

Materials and Methods

Supplementary Figure 1-13 with their legends

Supplementary Table 1- 6

**Materials and methods**

**Blood pressure measurement**

Heart rate and blood pressure (BP) were measured in the last week of Ang Ⅱ infusion using a computerized, noninvasive tail-cuff system (Kent Scientific Corporation, Torrington, CT, USA) as described previously^1^. In brief, the mice were habituated to the device daily for 1 week. For BP measurements, the mice were placed in a tail-cuff restrainer over a warmed surface. Twenty consecutive systolic BP measurements were taken, and the last ten readings per mouse were recorded and averaged.

**Ultrasound imaging**

Vascular ultrasonographic analyses were performed using a Vevo 2100 High Resolution Imaging System (Visual-Sonics) equipped with an MS-400 30 MHz transducer. Vascular ultrasonography was performed six times: the day before Ang Ⅱ infusion was initiated and after 3, 7, 14, 21 and 28 days of Ang Ⅱ infusion. The hair was removed using depilatory cream applied to the chest and stomach each time. Each mouse was anesthetized with isoflurane (3% induction and 1.5% maintenance) and placed on a heating pad in the supine position to maintain a body temperature of 37°C to minimize the confounding effects of fluctuating body temperatures. Images of the ascending and abdominal aortas were obtained. The maximal internal diameters were measured using the VEVO 2100 software by operators blinded to the experimental groups.

**Analyses and quantification of TAAs**

In the last week of the experiment, the mice underwent physiological assessments and were then sacrificed. After the aortas were dissected and separated from the surrounding connective tissue and weighed. Then, the aortas were photographed next to a ruler. These images were used to measure the outer diameter of the aortas by a researcher blinded to the group assignments. The maximum width of the aorta was analyzed using Image-Pro Plus software after adjusting the scale according to the ruler in the same picture. At least 3 measurements of the maximally expanded portion of the aorta segment for each mouse were averaged. For quantification of the aortic aneurysm, the aneurysm was defined as a 50% or greater increase in the external width of the aorta compared to that of the aortas from saline-infused mice, as previously described^2^.

**Bulk RNA sequencing (RNA-seq) analysis**

Mouse aortas were collected, and the adventitia was carefully removed. Total RNA was extracted from mouse aortic tissues using the GeneJet RNA Purification Kit (K0732, Thermo Scientific). The TruSeq RNA Library Prep Kit V2 (RS-122-2002, Illumina) was used to generate a sequencing library using 1 μg of RNA, according to the manufacturer’s instructions. All of the libraries were sequenced on a NextSeq500 sequencer (FC-404–2005, Illumina) using a 35 nt paired-end sequencing protocol.

Fastqc (<http://www.bioinformatics.babraham.ac.uk/projects/fastqc/>) was used to check the quality of the bulk RNA-seq reads. The reads were filtered using cutadapt and then mapped to genome mm10 using STAR. Only unique reads were kept for downstream analysis. The featureCounts function of the R package Rsubread was used to calculate read counts with in-build refseq gene annotation. Differentially expressed genes (DEGs) were detected by limma, and genes with a fold change greater than 1.5 and FDR less than 0.05 were considered significantly differentially expressed. ClusterProfiler^41^ was used for enrichment analysis.

**Histological analyses**

After the mice were sacrificed, aortas from the ascending aorta to the furcation of the common iliac artery were isolated without dissecting the surrounding connective tissue. The whole aortas were fixed with 4% paraformaldehyde-PBS for 48 h, and the thoracic aortas were then separated and embedded in paraffin. For the characterization of cross-sections, aortic sections were collected serially from the proximal to the distal aorta. Histology was determined in sections (5 µm each) taken from the aortas at intervals of 500 µm. Paraffin sections were used for Elastin Van Gieson (EVG) staining or immunostaining (immunohistochemical (IHC) staining and immunofluorescence (IF).

EVG staining was performed according to the manufacturer’s instructions (BA4083A, BASO, China). Briefly, slides were deparaffinized and incubated with potassium permanganate for 5 min. Next, the slides were blanched with oxalic acid for 5 min. Then, the slides were incubated with elastin solution overnight at 4°C followed by VG solution for 1 min at room temperature. The grade of elastin degradation was quantified by a researcher who was blinded to the group assignment based on the previously described criteria. Briefly, the elastic fiber quantification is a score for the entire section and the elastic fiber degradation grades are as follows: 1, no degradation; 2, mild elastin degradation; 3, severe elastin degradation; and 4, aortic rupture^3^.

Immunohistochemical (IHC) staining was performed according to the previously described method^4^. Briefly, slides were deparaffinized, and endogenous peroxidase activity was quenched with 3% hydrogen peroxide for 30 min. Next, nonspecific binding sites were blocked with goat serum at room temperature for 30 min. The slides were then incubated with diluted primary antibodies overnight at 4°C followed by biotinylated secondary antibodies for 30 min at 37°C and HRP-labelled streptavidin solution. Finally, the slides were stained with diaminobenzidine, counterstained with hematoxylin, and evaluated under a microscope. The primary antibodies used for IHC were mouse IL-1β (ab9722, 1:100 dilution; Abcam), mouse CD45 (ab10558, 1:200 dilution; Abcam), mouse Mac3 (550292, 1:50 dilution; BD), human SIRT6 (ab62739, 1:100 dilution; Abcam) and human MMP2 (ab37150, 4 μg/ml; Abcam). Species- and isotype-matched IgG were used in place of the primary antibody as a negative control.

Immunofluorescence (IF) staining was performed as previously described method^2^. First, the slides were deparaffinized and blocked with goat serum at room temperature for 1 h. The slides were then incubated at 4°C overnight with diluted primary antibodies, fluorescently conjugated secondary antibodies at 37°C for 45 min in the dark and DAPI for 5 min at room temperature in the dark. After the slides were rinsed with PBS, they were evaluated under a fluorescence microscope. The primary antibodies used were mouse CD45 (ab10558, 1:200 dilution; Abcam), mouse Mac3 (550292, 1:50 dilution; BD) and α-smooth muscle actin (A2547, 1:200 dilution; Sigma). All images were captured using the same settings. Images were analyzed using a quantitative digital image analysis system (Image-Pro Plus 6.0), as previously described^5, 6^.

**Senescence-associated β-galactosidase (SA-β-gal) staining**

Whole aortic tissues without the surrounding connective tissue were stained to determine SA-β-gal activity using a commercial kit (#K320-250; BioVision) according to the manufacturer’s instructions. Briefly, fresh tissues were fixed with Fixative Solution for 2 h at room temperature. Then, the tissues were washed with PBS 3 times and placed in Staining Solution Mix (470 µl of Staining Solution, 5 µl of Staining Supplement and 25 µl of 20 mg/ml X-gal in DMSO per 500 µl of Staining Solution Mix). The plate was covered and incubated overnight at 37°C. The tissues were washed with PBS, and the blue areas were considered SA-β-gal-positive.

To obtain aortic sections for SA-β-gal staining, aortic tissues were collected without removing the surrounding connective tissue to avoid damaging the aortic structure. Then, the sections were fixed, embedded, and sectioned as described in the histological analyses section. Moreover, nuclear Fast Red staining or IF was performed to visualize the nuclei.

**Sudan Black B (SBB) staining**

SBB staining was performed according to the manufacturer’s instructions (BA4088A, BASO, China) as previously described. First, tissue samples were dewaxed with xylene, dehydrated in 70% ethanol, and incubated with SBB solution for 5 min. Then, the tissues were embedded in 50% ethanol, transferred to and washed in distilled water, and counterstained with 0.1% Nuclear Fast Red for 10 min. Finally, the tissues were mounted with 40% glycerol/PBS mounting medium and evaluated under a microscope in a timely manner. Blue-black granules inside the cells indicate SBB staining.

**Cell Culture, Transfection and Drug Treatment**

Human vascular smooth muscle cells (VSMCs) were purchased from ScienCell (Cat No: 6110, ScienCell) and cultured in Smooth Muscle Cell Medium (SMCM, Cat No: 1101, ScienCell) supplemented with 100 U/ml penicillin, 100 μg/ml streptomycin, smooth muscle cell growth supplement (SMCGS, Cat. No. 1152, ScienCell), and 10% FBS. Primary mouse VSMCs were isolated from 2- to 3-month-old male mice and maintained in Dulbecco’s modified Eagle’s medium (DMEM) containing 10% FBS at 37 °C in a humidified atmosphere of 5% CO2 and 95% air as previously described^7^. Passages 3 to 8 of VSMCs at 70-80% confluence were used for experiments. The human VSMCs and mouse VSMCs were respectively treated with or without human (10139-HNAE, SinoBiological) and mouse (50101-MNAE, SinoBiological) recombinant IL-1β (100ng/ml) for 24 h. *Sirt6*-specific siRNA or scramble siRNA (Genepharma, Shanghai, China) was transiently transfected into human VSMCs using lipofectamine RNAiMAX (13778150, Thermo Fisher Scientific, Waltham, USA). The cells were subsequently cultured for 24 h, washed and incubated in 10% FBS-medium without virus and treated with 10^-6^ M angiotensin II (A9525, Sigma) or 1μg/μl Anakinra for 72 h. The primer sequences used are provided in Supplementary Table 5.

**Relative mRNA quantification**

Total RNA was extracted from aortic tissues using TRIzol reagent (#15596018, Thermo Fisher Scientific), according to the manufacturer’s instructions. Mouse aortas were collected, adventitia was carefully removed^2^, and the tunica media were processed for RNA purification and quantitative real-time PCR (qRT-PCR) analysis. First-strand cDNA was synthesized from 1 µg RNA using a reverse transcription system (TaKaRa) according to the manufacturer’s instructions. qRT-PCR was performed using AceQ qPCR SYBR Green Master Mix (Vazyme) on an Eppendorf Mastercycler machine with cDNA as a template. Primers were designed to span exon boundaries to avoid genomic DNA amplification, and the primer sequences are provided in Supplementary Table 4. Target mRNA expression was calculated relative to *β-actin*. And the delta-delta Ct formula was used to calculate gene expression^8^.

**Western blot analysis**

Mouse aortas were collected, and the adventitia was carefully removed. Western blotting was performed as previously described^6^. Aortic tissues were frozen in liquid nitrogen, and protein was extracted with RIPA lysis buffer (Beyotime Biotechnology) supplemented with a protease inhibitor cocktail (Roche) and phosphatase inhibitor (Roche). After sonication, the homogenates were centrifuged at 4°C for 15 min, and the supernatants were collected for western blot analysis. Then, the proteins were then separated using SDS-PAGE and electrotransferred to PVDF membranes (Millipore). Next, the membranes were then blocked with 5% fat-free milk/PBS and incubated with primary antibodies overnight at 4°C. Finally, the membranes were incubated with an HRP-conjugated secondary antibody and exposed to Pierce ECL Western Blot Substrate to assess protein expression. The primary antibodies used were human SIRT6 (12486, 1:1000 dilution; Cell Signaling Technology), human MMP2 (ab37150, 1:1000 dilution; Abcam), human GAPDH (AT0002, 1:5000 dilution; CMCTAG), human and mouse P21 (05-345, 1:1000; Millipore), human and mouse P53 (sc-73566, 1:1000 dilution; Santa Cruz), human IL-1β (ab9722, 1:1000 dilution; Abcam), and mouse Tubulin (AT0003, 1:5000 dilution; CMCTAG). The western blot bands were quantified densitometrically using ImageJ software, and the band density was calculated relative to the density of the tubulin or GAPDH band.

**Chromatin immunoprecipitation (ChIP) assay**

As described in previous research^2^, freshly isolated mouse aortas were carefully stripped out of the adventitial layer, chopped into small pieces and crosslinked with 1% formaldehyde at room temperature for 20 min. Then, the reaction was quenched with glycine (final concentration of 125 mM). After the samples were washed with cold PBS, the tissue pieces were ground with cell lysis buffer, and the mixture was incubated at room temperature for 15 min and at 30°C for 15 min. Nucleus/chromatin preparation buffers I and II were used to isolate the nuclei. Next, the nuclear pellet was washed with cold PBS, resuspended in SDS lysis buffer and sonicated to generate chromatin fragments with an average size of 200–500 bp. Chromatin lysates were cleared by centrifugation and subjected to immunoprecipitation with ChIP-grade antibodies against SIRT6 (ab62739; Abcam), H3 (ab1791; Abcam), H3K9ac (ab10812; Abcam), H3K56ac (A-4026; Epigentek), IRF8 (5268, Cell Signaling Technology) and normal rabbit IgG (2729, Cell Signaling Technology). The DNA-protein complex was precipitated with protein G Dynabeads (Invitrogen) and reverse crosslinked using Proteinase K and RNase A. The DNA was purified and analyzed using qRT-PCR. Specific primers were designed to amplify the *Il1b* promoter. The primer sequences used are provided in Supplementary Table 6.


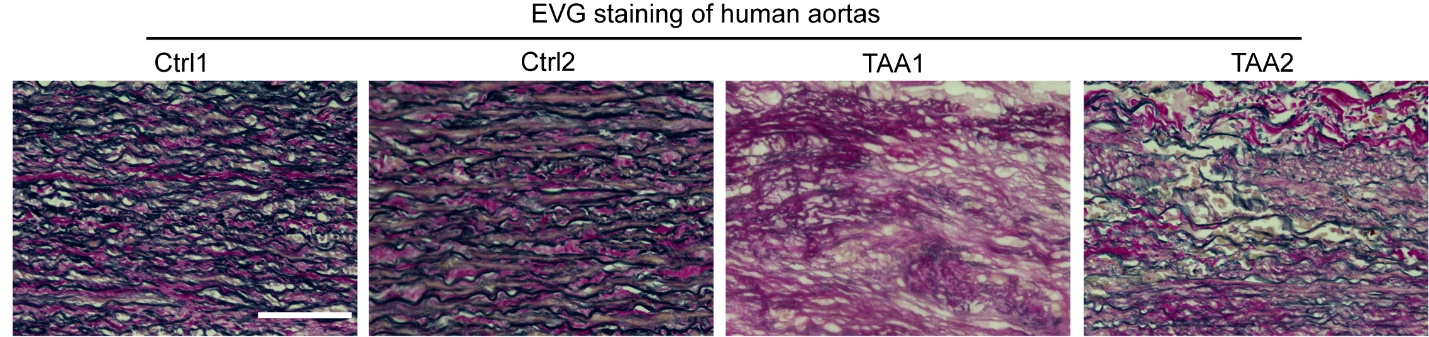


**Supplementary Fig 1. Increased elastin disorganization in human sporadic TAA samples.** EVG staining of elastin in human sporadic TAA and control samples (scale bar: 200 μm).


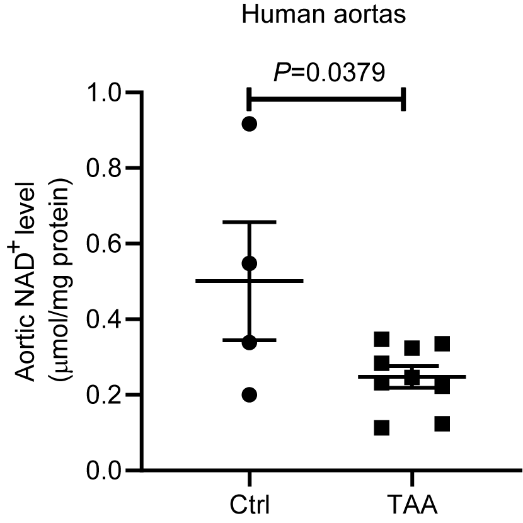


**Supplementary Fig 2. Reduced NAD^+^ levels in sporadic human TAA samples.** NAD^+^ level in aortas of sporadic TAA patients (n=9) and control samples (n=4).


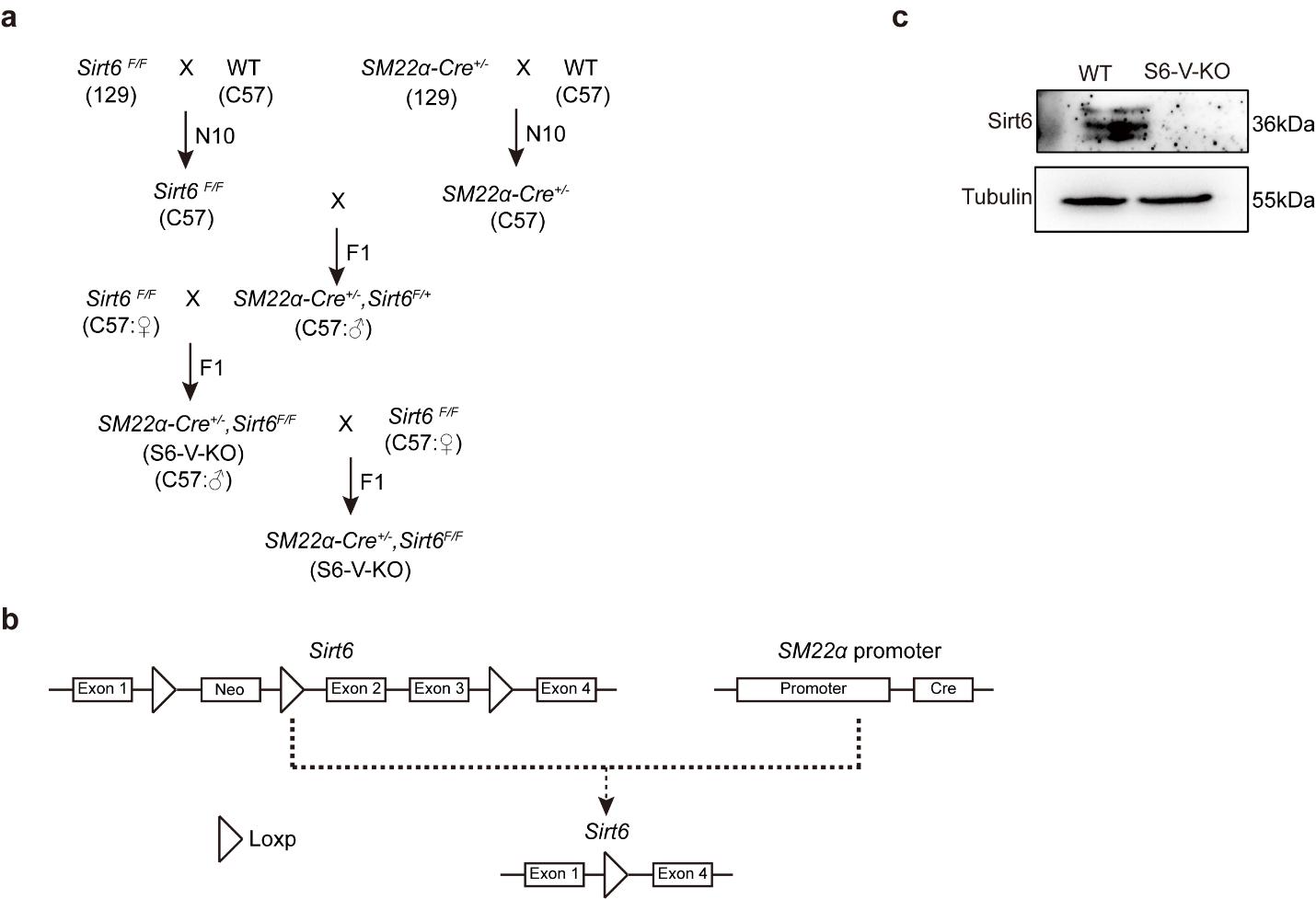


**Supplementary Fig 3. Induction and assessment of selective knockout of *Sirt6* in mouse VSMCs.** **a.** Breeding strategy used to generate S6-V-KO mice. F, filial generations; N, number of backcrossed generations. **b.** Design of a *Cre/LoxP* system to delete the SIRT6 deacetylase domain, which is encoded by exons 2 and 3, in mouse SMCs. Neo, neomycin. **c.** Western blot analysis of SIRT6 expression in the aortas of WT and S6-V-KO mice.


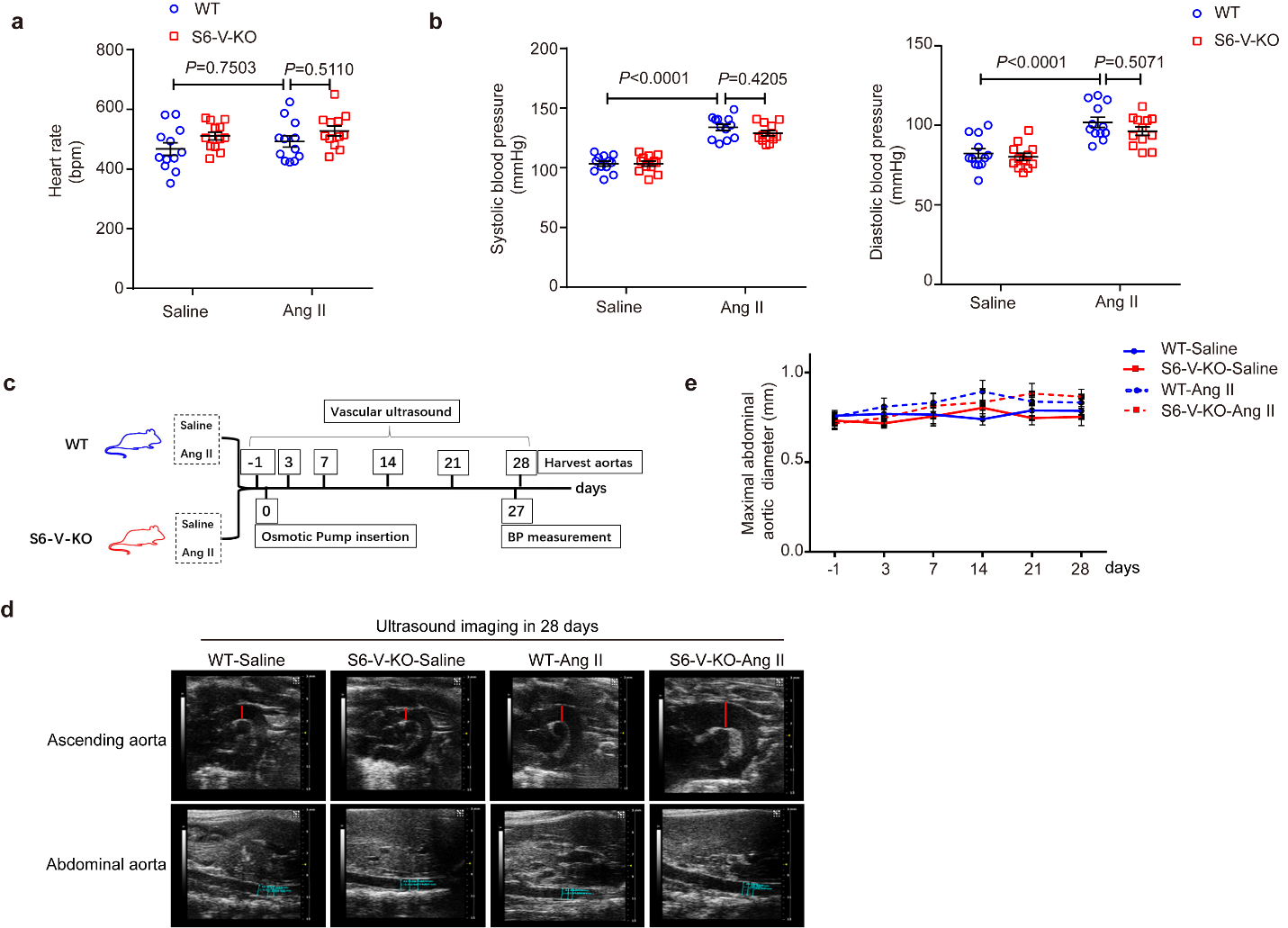


**Supplementary Fig 4. Heart rates and blood pressures of WT and S6-V-KO mice infused with saline or Ang Ⅱ. a.** Heart rates of WT and S6-V-KO mice infused with saline or Ang Ⅱ for 28 days (n=12 mice/group). **b.** The systolic and diastolic blood pressure of WT and S6-V-KO mice infused with saline or Ang Ⅱ (n=12 mice/group). **c.** Experimental design. All mice were infused with saline or Ang Ⅱ for 28 days. Blood pressure was measured and vascular ultrasound was performed at the time points shown in the figure. **d.** Representative ultrasound imaging of WT and S6-V-KO mice ascending and abdominal aortas after saline or Ang II infusion for 28 days. **e.** The maximal internal diameter of abdominal aortas in the indicated groups at Days -1, 3, 7, 14, 21 and 28 (n=5-7 mice/group).


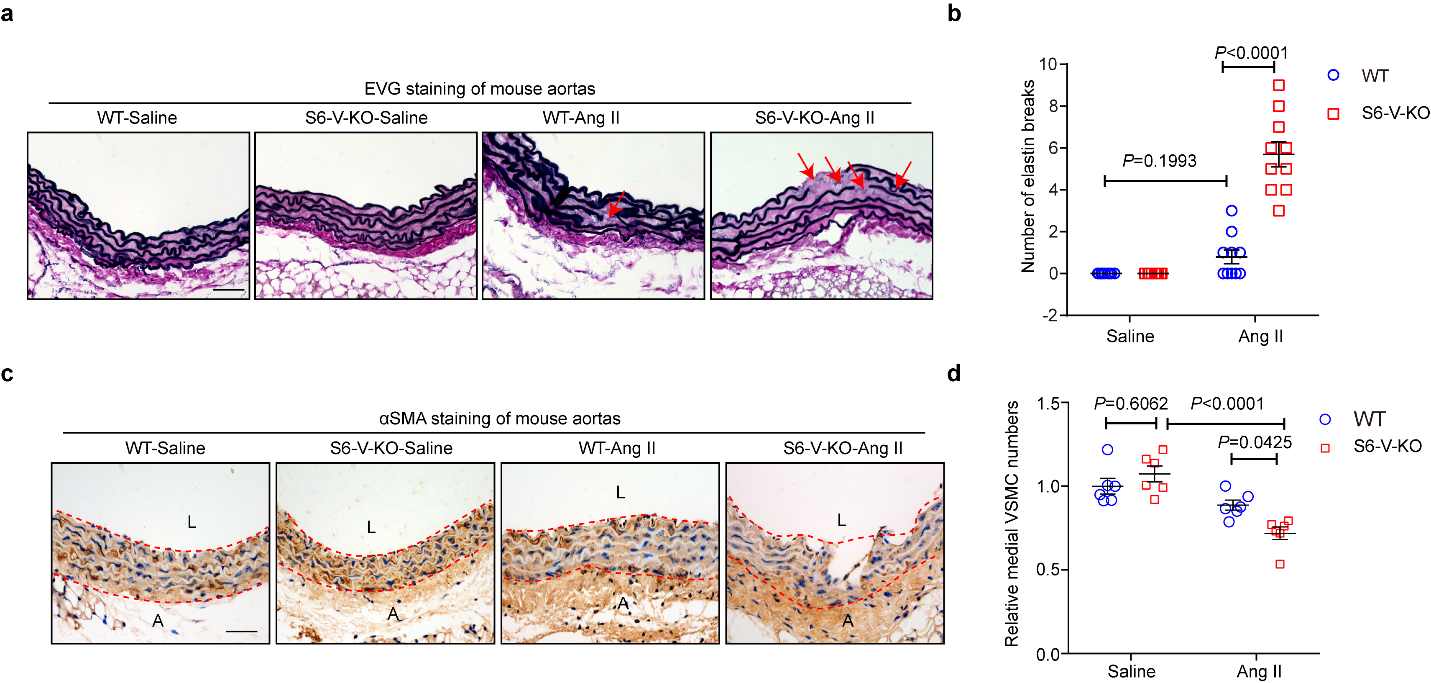


**Supplementary Fig 5.** ***Sirt6* deletion in VSMCs promotes TAA severity after Ang Ⅱ infusion for 28 days.** **a.** Representative images of EVG staining in the indicated groups (scale bar:100μm), **b.** Quantitative results of EVG staining through calculating the number of elastin fiber breakage (n=10 mice/group), **c.** IHC staining of αSMA in the ascending aortas of mice in the indicated groups. L, lumen; A, adventitia (scale bar: 100 µm). **d.** Quantification of αSMA-positive VSMC number (n=6 mice/group).

**
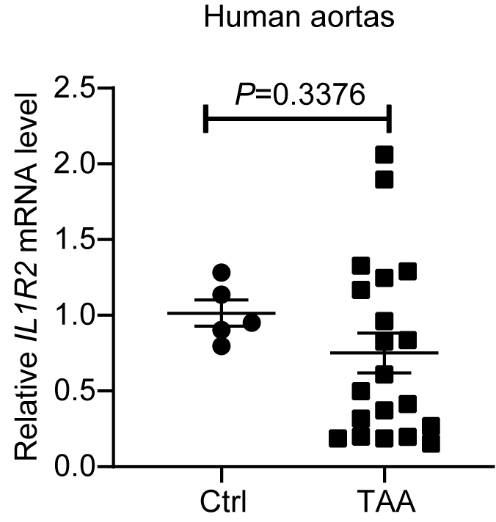
**

**Supplementary Fig 6.** mRNA levels of *IL1R2* in human control thoracic aortas (n=5) and TAA samples (n=20).


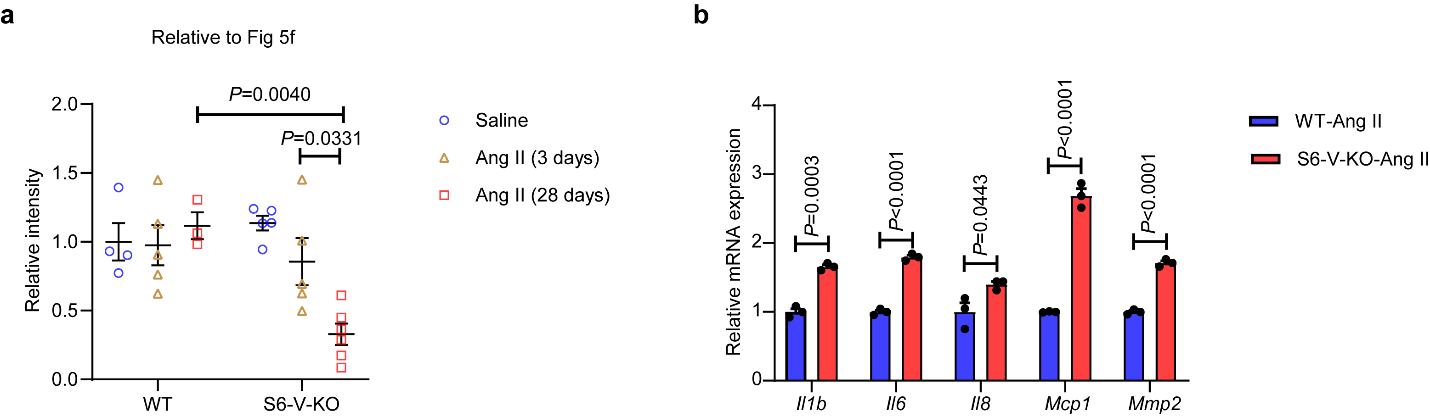


**Supplementary Fig 7.** **a.** The quantification of αSMA related to Figure 5f (n=3-6 mice/group). **b.** Relative mRNA level of inflammation-related factors and *Mmp2* in the Ang II-treated WT and S6-V-KO VSMCs for 72h (n=3).


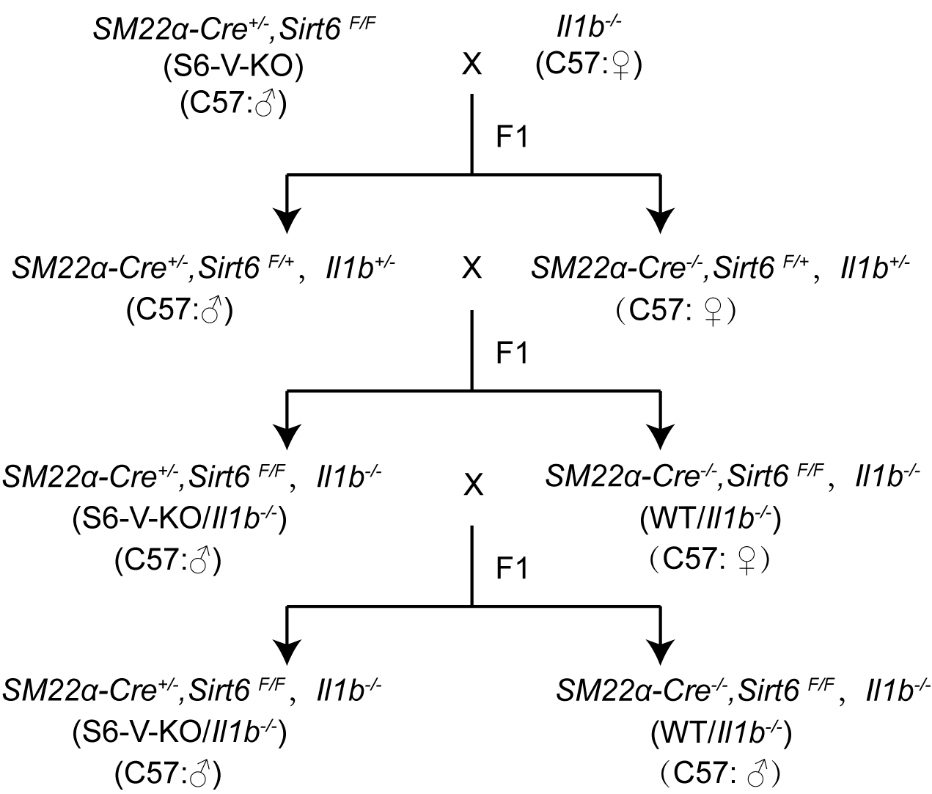


**Supplementary Fig 8.** **Breeding strategy for generating the S6-V-KO/*Il1b^-/-^* and WT /*Il1b^-/-^* mice.**

**
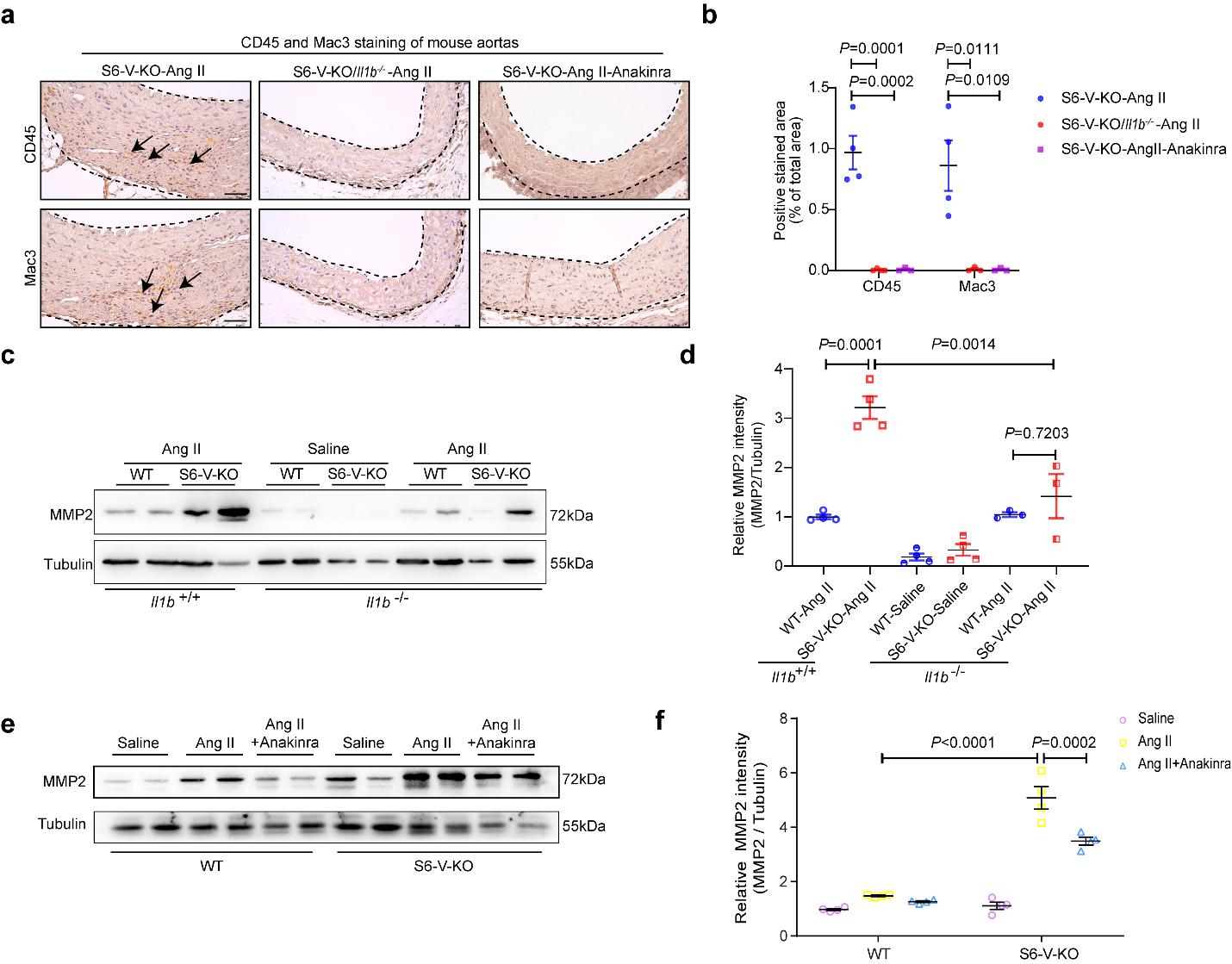
**

**Supplementary Fig 9. Genetic and Pharmacological inhibition of IL-1β alleviates Ang Ⅱ-induced inflammatory cell infiltration and MMP2 expression.** **a.** Representative images of IHC staining of leukocytes (CD45, top) and macrophages (Mac3, bottom) in the thoracic aortas of mice in the indicated groups (scale bar: 50 µm). The arrows represent positive staining areas. **b.** Statistical analysis of the CD45 and Mac3 positive area percentage in mouse aortas (n=3-4 mice/group). **c.** The protein levels of MMP2 and Tubulin in the aortic homogenates from the indicated groups were measured by western blotting. **d.** The quantification of MMP2 protein expression upon genetic inhibition of *Il1b* (n=4 mice/group). **e.** The protein levels of MMP2 and Tubulin in the aortic homogenates from the indicated groups were measured by western blotting. **f.** The quantification of MMP2 protein expression upon pharmacological inhibition of IL-1β signaling pathway (n=4 mice/group).

**
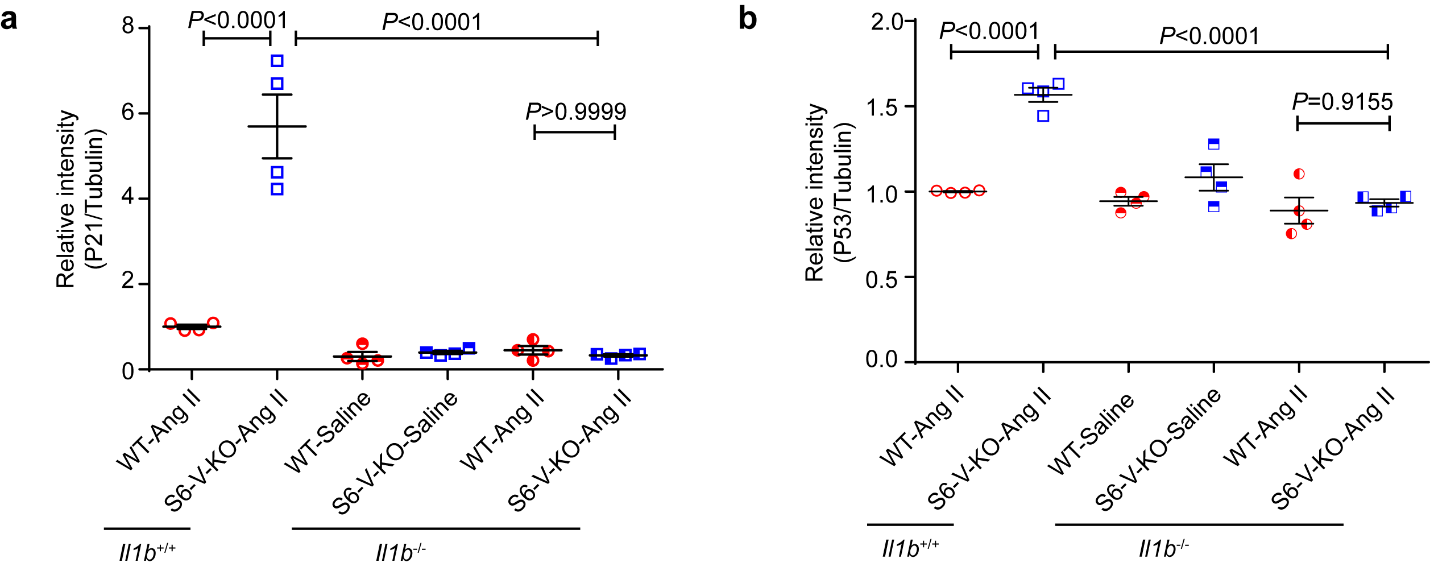
**

**Supplementary Fig 10.**  **Genetic inhibition of *Il1b* decreases the expression of senescence-associated molecules.** **a-b.** The quantification of P53 and P21 protein expression upon genetic inhibition of *Il1b* (n=4 mice/group).


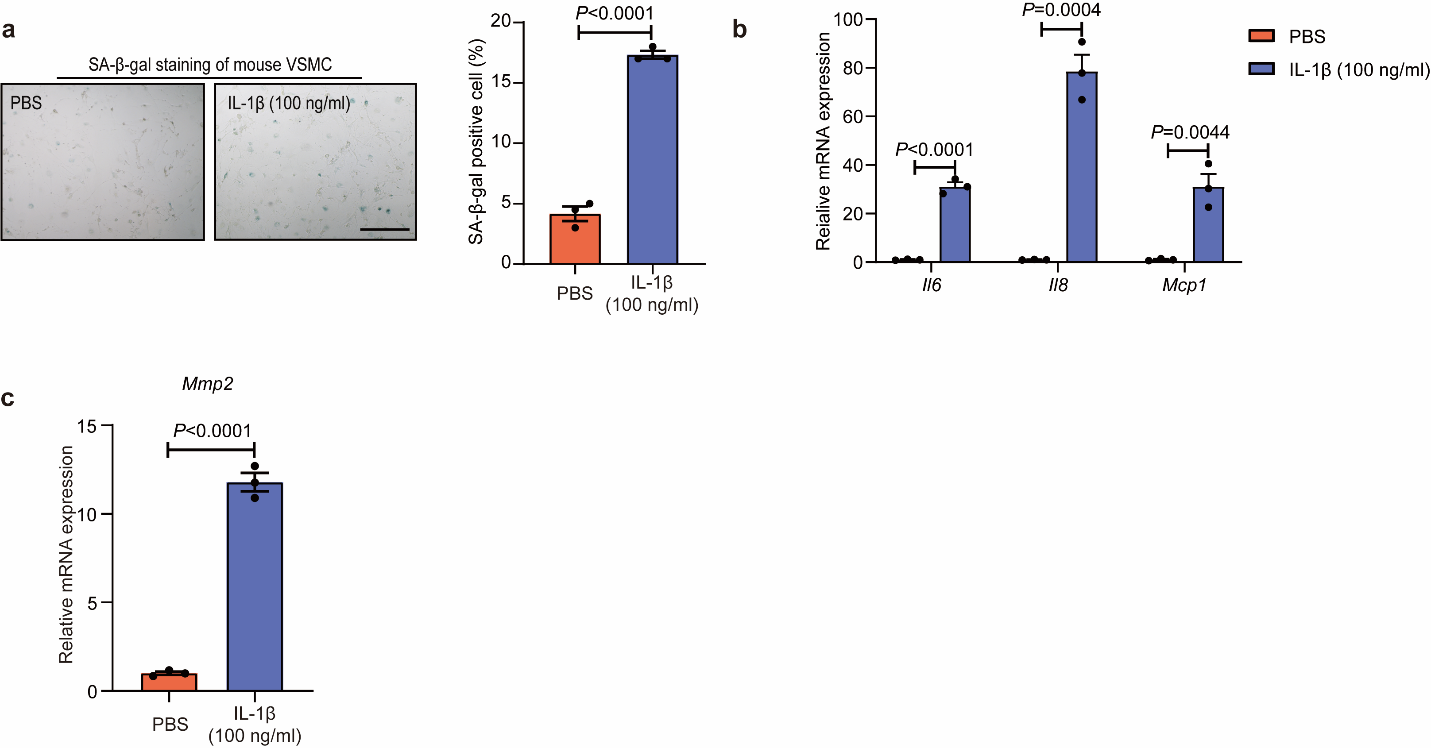


**Supplementary Fig 11. IL-1β induces VSMCs inflammation and senescence *in vitro*.** **a.** Representative images of SA-β-gal-stained mouse VSMCs treated with or without IL-1β (100 ng/ml) and densitometric analysis of SA-β-gal staining positive cells. Blue-stained cells were considered senescent. Data are presented as the mean±SEM of 3 independent experiments (scale bar: 500μm). **b.** mRNA levels of inflammatory genes in mouse VSMCs in the indicated groups (n=3). **c.** mRNA level of *Mmp2* in mouse VSMCs treated with or without IL-1β (100 ng/ml) (n=3).


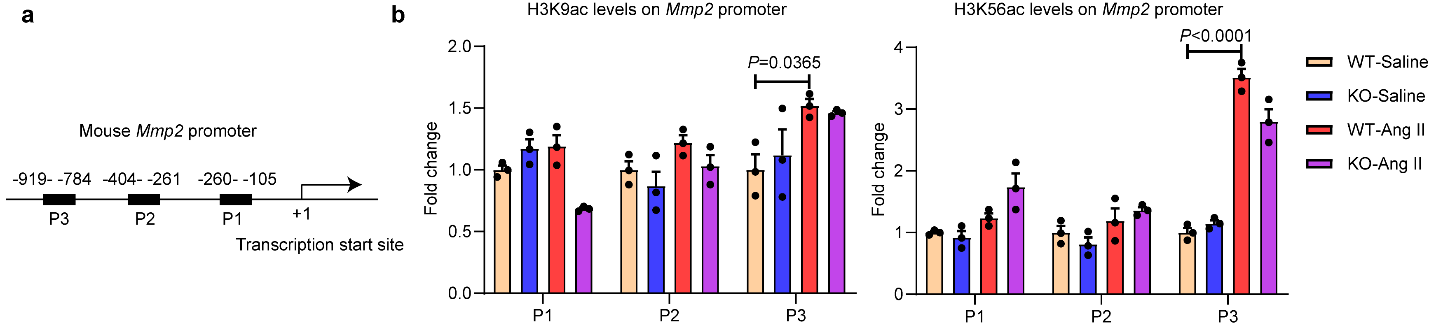


**Supplementary Fig 12**. ***Sirt6* deficiency doesn’t increase H3K9ac and H3K56ac on the *Mmp2* promoters.** **a.** Diagram showing the designed primers for mouse *Mmp2* promoter. **b.** ChIP of H3K9ac and H3K56ac at the *Mmp2* promoter in the aortas of WT and S6-V-KO mice after saline or Ang Ⅱ infusion for 28 days (n=3 mice/group). Chromatin was immunoprecipitated with normal rabbit IgG or antibodies against H3K9ac, H3K56ac and H3, and precipitated genomic DNA was analyzed by qPCR using different primers for the different regions of the *Mmp2* promoter.


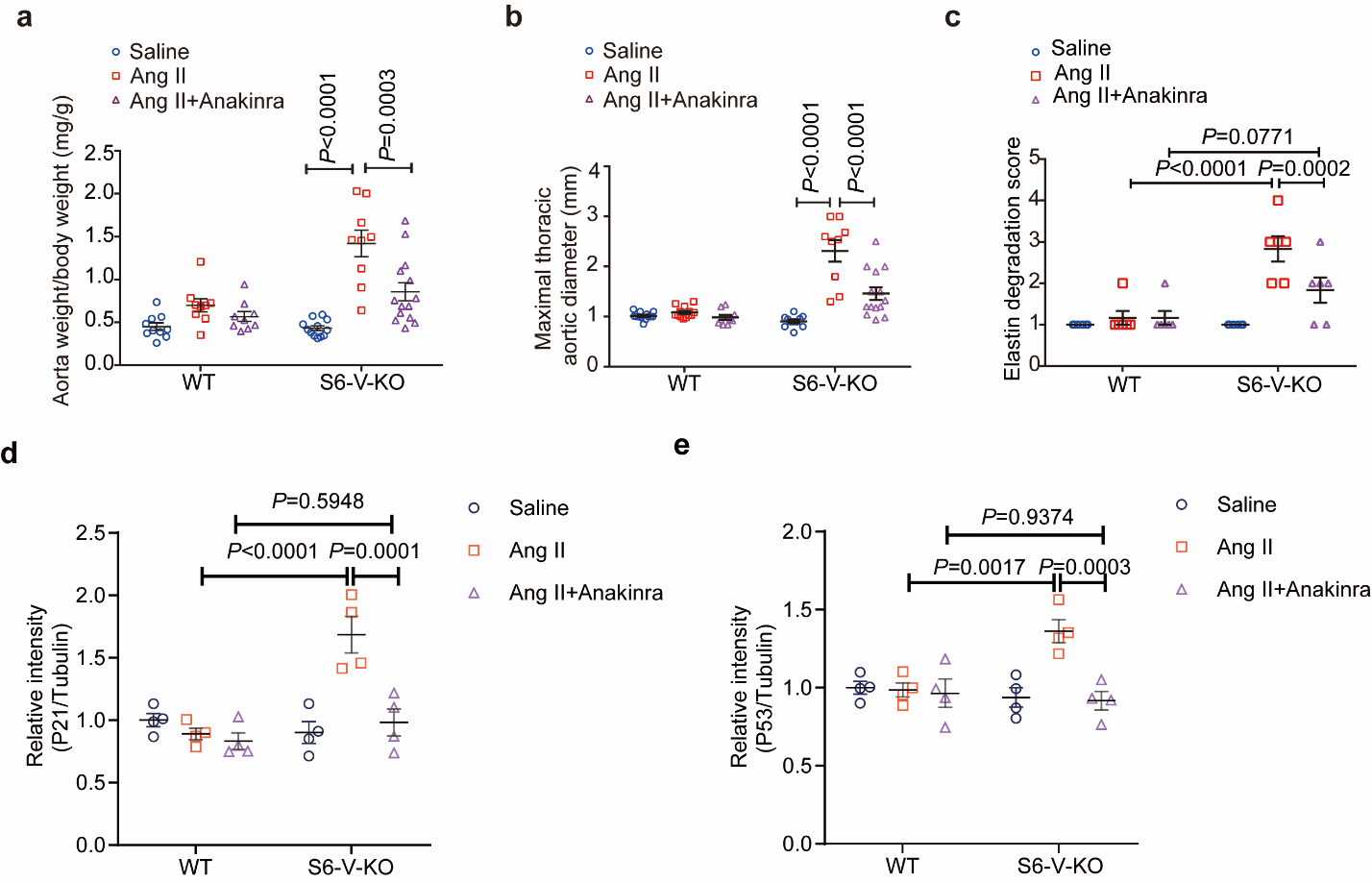


**Supplementary Fig 13. Pharmacological inhibition of IL-1β alleviates Ang Ⅱ-induced TAA severity upon *Sirt6* deficiency. a.** The ratio of aortic weight to body weight (n=9-14 mice/group). **b.** Maximal thoracic aortic outer diameter (n=9-14 mice/group) in the indicated group. **c.** Semiquantitative analysis of elastin degradation (n=6 mice/group). **d-e.** The quantification of P21 and P53 protein expression upon pharmacological inhibition of IL-1β signaling pathway (n=4 mice/group).

**Supplementary Tables**

**Supplementary Table 1. The information of clinical samples.**

| Number | Age | Gender | Clinical diagnosis |
| --- | --- | --- | --- |
| *Ctrl-1 | 47 | Male | Cerebral hemorrhage |
| *Ctrl-2 | 61 | Male | Lung cancer |
| *Ctrl-3 | 73 | Male | Myocardial infarction |
| *Ctrl-4 | 73 | Female | Cerebral aneurysm |
| Ctrl-5 | 22 | Male | Pericardial tumor |
| Ctrl-6 | 71 | Female | Thymic carcinoma |
| *TAA-1 | 65 | Male | TAA |
| *TAA-2 | 63 | Female | TAA |
| *TAA-3 | 47 | Male | TAA |
| *TAA-4 | 61 | Male | TAA |
| TAA-5 | 73 | Male | TAA |
| TAA-6 | 47 | Male | TAA |
| TAA-7 | 64 | Male | TAA |
| TAA-8 | 55 | Male | TAA |
| TAA-9 | 54 | Female | TAA |
| TAA-10 | 68 | Female | TAA |
| TAA-11 | 67 | Male | TAA |
| TAA-12 | 52 | Female | TAA |
| TAA-13 | 44 | Male | TAA |
| TAA-14 | 69 | Female | TAA |
| TAA-15 | 41 | Male | TAA |
| TAA-16 | 65 | Female | TAA |
| TAA-17 | 55 | Female | TAA |
| TAA-18 | 71 | Male | TAA |
| TAA-19 | 61 | Male | TAA |
| TAA-20 | 45 | Male | TAA |

* These samples were also used for Western blot in Fig.1a, and Fig. 3j.

**Supplementary** **Table 2. Aneurysm formation and rupture in Ang Ⅱ-infused mice.**

**Supplementary Table 2.1. SMC-specific *Sirt6* deficiency promotes TAA formation in Ang Ⅱ- induced mouse models (Ang Ⅱ:1.44 mg/kg/d).**

Ang Ⅱ:1.44 mg/kg/d

| Group | Total number | Death due to  TA rupture | Death due to  AA rupture | Survived mice number | TAA in  survived mice | AAA in survived mice |
| --- | --- | --- | --- | --- | --- | --- |
| S6-V-KO-Ang Ⅱ | 51 | 36（36/51;70.59%） | 4  （4/51;7.84%） | 11 | 7  （7/11;63.63%） | 1  （1/11;10%） |
| WT- Ang Ⅱ | 39 | 2 （2/39;5.13%） | 0  (0/39;0%) | 37 | 0  (0/37;0%) | 0  (0/37;0%) |

**Supplementary Table 2.2.** **SMC-specific *Sirt6* deficiency promotes TAA formation in Ang Ⅱ- induced mouse models (Ang Ⅱ:0.72 mg/kg/d).**

Ang Ⅱ:0.72 mg/kg/d

| Group | Total number | Death due to TA rupture | Death due to AA rupture | Survived mice number | TAA in survived mice | AAA in survived mice |
| --- | --- | --- | --- | --- | --- | --- |
| S6-V-KO-Ang Ⅱ | 49 | 27  (27/49;55.10%) | 0  (0/49;0%) | 22 | 13  (13/22;59.10%) | 3  (3/22;13.64%) |
| WT- Ang Ⅱ | 31 | 2  (2/31;6.45%) | 1  (1/31;3.23%) | 28 | 0  (0/28;0%) | 1  (1/28;3.57%) |

**Supplementary Table 2.3. Genetic inhibition of *Il1b* alleviates Ang Ⅱ-induced TAAs.**

Ang Ⅱ: 0.72 mg/kg/d

| Group | Total number | Death due to TA rupture | Death due to AA rupture | Survived mice number | TAA in  survived mice | AAA in survived mice |
| --- | --- | --- | --- | --- | --- | --- |
| S6-V-KO / *Il1b ^+/+^*  -Ang Ⅱ | 35 | 21  (21/35;60%) | 1  (1/35;2.86%) | 13 | 7  (7/13;53.85%) | 0  (0/13;0%) |
| S6-V-KO / *Il1b ^-/-^*  -Ang Ⅱ | 35 | 14  (14/35;40%) | 0  (0/35;0%) | 21 | 5  (5/21;23.81%) | 2  (2/21;9.52%) |
| WT**/** *Il1b ^+/+^*  - Ang Ⅱ | 19 | 1  (1/19;5.26%) | 2  (2/19;10.52%) | 16 | 0  (0/16;0%) | 1  (1/16;6.25%) |
| WT / *Il1b ^-/-^*  -Ang Ⅱ | 22 | 1  (1/22;4.54%) | 1  (1/22;4.54%) | 20 | 0  (0/20;0%) | 0  (0/20;0%) |

**Supplementary Table 2.4. Pharmacological inhibition of IL-1β alleviates Ang Ⅱ-induced TAAs.**

Ang Ⅱ: 0.72 mg/kg/d

| Group | Total number | Death due to TA rupture | Death due to AA rupture | Survived mice number | TAA in survived mice | AAA in survived mice |
| --- | --- | --- | --- | --- | --- | --- |
| S6-V-KO-Ang Ⅱ | 30 | 17  (17/30;56.67%) | 1  (1/30;3.33%) | 12 | 6  (6/12;50%) | 2  (2/12;16.67%) |
| S6-V-KO-Ang Ⅱ - Anakinra | 29 | 9  (9/29;31.03%) | 0  (0/29;0%) | 20 | 5  (5/20;25%) | 2  (2/20;10%) |
| WT- Ang Ⅱ | 22 | 1  (1/22;4.54%) | 1  (1/22;4.54%) | 20 | 0  (0/20;0%) | 1  (1/20;5%) |
| WT- Ang Ⅱ- Anakinra | 13 | 1  (1/13;7.69%) | 0  (0/13;0%) | 12 | 0  (0/12;0%) | 0  (0/12;0%) |

**Supplementary Table 3. Primers applied in PCR genotyping on mouse genomic DNA.**

| Gene | Primer | Primer sequence (5'-3') | Primer type |
| --- | --- | --- | --- |
| *SM22α-Cre* | oIMR7055 | GGCCCAGGGGTTGTCAAAATAGTC | Common |
|  | oIMR7056 | CTCCTCCAGCTCCTCGTCATACTTC | Wide type reverse |
|  | oIMR7057 | CGCCGCATAACCAGTGAAACAG | Mutant reverse |
| *Sirt6-Loxp* | P1 | GCTAATGGGAACGAGACCAA | Forward |
|  | P2 | ACCCACCTCTCTCCCCTAAA | Reverse |
| *Il1b^-/-^* | P1 | ACCTATACAACGGCTCCTC | Common |
|  | P2 | TGACGAACGTGTACATCGAC | Mutant reverse |
|  | P3 | GTGGCTGTGGTAAATGAAA | Wide type reverse |

**Supplementary Table 4. Primers applied in qRT-PCR.**

| Gene | Forward (5'-3') | Reverse (5'-3') |
| --- | --- | --- |
| Mouse-*Actb* | GGCTGTATTCCCCTCCATCG | CCAGTTGGTAACAATGCCATG |
| Mouse-*Il1b* | GCAACTGTTCCTGAACTCAACT | ATCTTTTGGGGTCCGTCAACT |
| Mouse-*Il6* | CTGCAAGAGACTTCCATCCAG | AGTGGTATAGACAGGTCTGTTGG |
| Mouse-*Il8* | TGTTGAGCATGAAAAGCCTCTAT | AGGTCTCCCGAATTGGAAAGG |
| Mouse-*Mcp1* | TTAAAAACCTGGATCGGAACCAA | TTAAAAACCTGGATCGGAACCAA |
| Mouse-*Cdkn1a* | CCTGGTGATGTCCGACCTG | CCATGAGCGCATCGCAATC |
| Mouse-*Tp53* | GCGTAAACGCTTCGAGATGTT | TTTTTATGGCGGGAAGTAGACTG |
| Human-*Actb* | CATGTACGTTGCTATCCAGGC | CTCCTTAATGTCACGCACGAT |
| Human-*SIRT6* | CCCACGGAGTCTGGACCAT | CTCTGCCAGTTTGTCCCTG |
| Human-*MMP2* | CCCACTGCGGTTTTCTCGAAT | CAAAGGGGTATCCATCGCCAT |
| Human-*IL1β* | ATGATGGCTTATTACAGTGGCAA | GTCGGAGATTCGTAGCTGGA |
| Human-*CDKN1A* | TGTCCGTCAGAACCCATGC | AAAGTCGAAGTTCCATCGCTC |
| Human-*TP53* | GAGGTTGGCTCTGACTGTACC | TCCGTCCCAGTAGATTACCAC |
| Human-*IL1R2* | TCCTGCCGTTCATCTCATACC | CATCGTGTACGAGTAAGTGAGTG |

**Supplementary Table 5. Primer sequences used in *SIRT6* interference.**

| Gene | Forward (5'-3') | Reverse (5'-3') |
| --- | --- | --- |
| Human-*SIRT6* | UCAUGACCCGGCUCAUGAATT | UUCAUGAGCCGGGUCAUGATT |
| Negative control | UUCUCCGAACGUGUCACGUTT | ACGUGACACGUUCGGAGAATT |

**Supplementary Table 6. Primer sequences used in ChIP assay.**

| Amplified site on genome | Primer sequence(sense/anti-sense,5'-3') |
| --- | --- |
| Mouse *Il1b* promoter 1 | ATGTGCGGAACAAAGGTAGG |
| -515 to -300 | CCTGACCCACACAAGGAAGT |
| Mouse *Il1b* promoter 2 | TTCACAGGGTCCACATTTCA |
| -1841 to -1648 | CAGGCCCCTAGTTACCCTTC |
| Mouse *Il1b* promoter 3 | GGGGTCTGTTCTTTCCCTCT |
| -2998 to -2819 | AGGCTGATTTCTCCCCAAGT |
| Mouse *Mmp2* promoter 1 | CAAGCCAAGGGATAGAGGACA |
|  | GCAAGGATAATCTGGAAAGGAGG |
| Mouse *Mmp2* promoter 2 | ACA ACTCAGAAGTCACATCGTCC |
|  | GGTTTCACTGGTGGTCCTCAAT |
| Mouse *Mmp2* promoter 3 | CTTGAGTGGCTCTATGGCTGAT |
|  | TGCCTTTGGAGGGATACTGG |

**References**

1. Li L*, et al.* SIRT1 acts as a modulator of neointima formation following vascular injury in mice. *Circ Res* **108**, 1180-1189 (2011).

2. Liu Y*, et al.* Calorie restriction protects against experimental abdominal aortic aneurysms in mice. *The Journal of experimental medicine* **213**, 2473-2488 (2016).

3. Satoh K*, et al.* Cyclophilin A enhances vascular oxidative stress and the development of angiotensin II-induced aortic aneurysms. *Nat Med* **15**, 649-656 (2009).

4. Yang K*, et al.* Prevention of aortic dissection and aneurysm via an ALDH2-mediated switch in vascular smooth muscle cell phenotype. *Eur Heart J* **41**, 2442-2453 (2020).

5. Chen HZ*, et al.* Age-Associated Sirtuin 1 Reduction in Vascular Smooth Muscle Links Vascular Senescence and Inflammation to Abdominal Aortic Aneurysm. *Circ Res* **119**, 1076-1088 (2016).

6. Tang X*, et al.* SIRT2 Acts as a Cardioprotective Deacetylase in Pathological Cardiac Hypertrophy. *Circulation* **136**, 2051-2067 (2017).

7. Ishida T, Ishida M, Suero J, Takahashi M, Berk BC. Agonist-stimulated cytoskeletal reorganization and signal transduction at focal adhesions in vascular smooth muscle cells require c-Src. *J Clin Invest* **103**, 789-797 (1999).

8. Livak KJ, Schmittgen TD. Analysis of relative gene expression data using real-time quantitative PCR and the 2(-Delta Delta C(T)) Method. *Methods* **25**, 402-408 (2001).
